# Checkpoint Imbalance in Primary Glomerulopathies: Comparative Insights into IgA Nephropathy and Membranoproliferative Glomerulonephritis

**DOI:** 10.3390/cells14191551

**Published:** 2025-10-03

**Authors:** Sebastian Mertowski, Paulina Mertowska, Milena Czosnek, Iwona Smarz-Widelska, Wojciech Załuska, Ewelina Grywalska

**Affiliations:** 1 Department of Experimental Immunology, Medical University of Lublin, Chodźki 4a Street, 20-093 Lublin, Poland; paulina.mertowska@umlub.edu.pl (P.M.); ewelina.grywalska@umlub.edu.pl (E.G.); 2 Student Research Group of Experimental Immunology, Medical University of Lublin, Chodźki 4a Street, 20-093 Lublin, Poland; 3 Department of Nephrology, Cardinal Stefan Wyszynski Provincial Hospital in Lublin, Al. Kraśnicka Street, 20-718 Lublin, Poland; 4 Department of Nephrology, Medical University of Lublin, 8 Jaczewskiego Street, 20-954 Lublin, Poland; wojciech.zaluska@umlub.edu.pl

**Keywords:** immune system, immune checkpoint, glomerulonephritis, IgAN, MPGN, PD-1, CTLA-4, CD200R

## Abstract

Introduction: Primary glomerulopathies are immune-driven kidney diseases. IgA nephropathy (IgAN) and membranoproliferative glomerulonephritis (MPGN) are prevalent entities with a risk of chronic progression. Immune checkpoints, such as PD-1/PD-L1, CTLA-4/CD86, and CD200R/CD200, regulate activation and tolerance in T, B, and NK cells, and also exist in soluble forms, reflecting systemic immune balance. Objective: To compare immune checkpoint profiles in IgAN and MPGN versus healthy volunteers (HV) through surface expression, soluble serum levels, and PBMC transcripts, with attention to sex-related differences and diagnostic value assessed by ROC curves. Materials and Methods: Ninety age-matched subjects were studied: IgAN (*n* = 30), MPGN (*n* = 30), HV (*n* = 30). Flow cytometry evaluated checkpoint expression on CD4+/CD8+ T cells, CD19+ B cells, and NK cells. ELISA quantified sPD-1, sPD-L1, sCTLA-4, sCD86, sCD200, sCD200R; PBMC transcript levels were assessed. Group comparisons, sex stratification, and ROC analyses were performed. Results: Lymphocyte distributions were preserved, but IgAN patients showed anemia and impaired renal function, while MPGN patients had greater proteinuria and dyslipidemia. GN patients displayed increased PD-1/PD-L1 and CD200R/CD200, with reduced CTLA-4/CD86, compared to HV. Serum analysis revealed elevated sPD-1, sPD-L1, sCD200, sCD200R and decreased sCTLA-4, sCD86. PBMC transcripts paralleled these trends, with PD-1/PD-L1 mainly increased in MPGN. Sex had minimal impact. ROC analyses showed strong GN vs. HV discrimination by CD19^+^CTLA-4^+^, PD-1/PD-L1, and CD200/CD200R, but limited ability to separate IgAN from MPGN. Conclusions: IgAN and MPGN share a sex-independent checkpoint signature: PD-1/PD-L1 and CD200R/CD200 upregulation with CTLA-4/CD86 downregulation. CD19^+^, CTLA-4^+^, and soluble PD-1/PD-L1/CD200(R) emerge as promising biomarkers requiring further validation.

## 1. Introduction

Primary glomerulopathies (GN) belong to a diverse group of glomerular diseases characterized by distinct histopathological features, clinical course, and prognosis [1,2,3]. However, a common element in their pathogenesis is the significant involvement of immune system dysregulation [4,5,6,7]. Among them, IgA nephropathy (IgAN) and mesangial-capillary glomerulonephritis (MPGN) constitute fundamental clinical entities due to their prevalence, chronic nature, and risk of progression to end-stage renal disease [8,9,10,11,12,13,14].

IgA nephropathy is the most common GN worldwide. It develops as a result of overproduction of abnormally glycosylated IgA1 (Gd-IgA1), the formation of autoantibodies against its epitopes, and the formation of circulating immune complexes with high affinity for the mesangium. These complexes initiate local complement activation, primarily via the alternative and lectin pathways, leading to mesangial cell damage, proliferation, and fibrosis [15,16,17,18].

MPGN, on the other hand, is a heterogeneous entity whose common denominator is a histological pattern characterized by thickening of basement membranes, mesangial cell proliferation, and the formation of a so-called “double contour.” Currently, two forms are distinguished: the immunocomplex-associated form (IC-MPGN), in which immune complex deposition plays a key role, and forms associated with dysregulation of the alternative complement pathway (C3-glomerulopathies). In both cases, the common denominator is the chronic stimulation of the immune response, the regulation of which depends on the interaction of humoral and cellular mechanisms [19,20,21,22,23].

The immune system plays a key role in the pathogenesis of GN, as it co-determines the initiation, maintenance, and resolution of inflammation in the glomeruli and the transition from transient changes to chronic kidney damage.

In this context, immune checkpoints play a crucial role as a link between humoral mechanisms (immune complexes, complement) and cellular regulation, coordinating the overall inflammatory response [24,25,26]. By setting activation and inhibition thresholds, they determine whether the reaction will subside or become chronic [27,28]. For example, the PD-1/PD-L1 axis is a key inhibitory system that, through ligand binding, limits effector programs and modulates the duration and severity of inflammation [29,30,31,32,33]. Furthermore, CTLA-4 acts in the early phases of the response as a “gate” competing for costimulatory signals, shaping the scope and quality of lymphocyte activation [34,35,36,37,38]. Another layer of control is provided by the CD200/CD200R axis, which dampens excessive activation and promotes the maintenance of tissue tolerance; its disruption promotes chronic inflammation [39,40,41].

Soluble forms of checkpoints (sPD-1, sPD-L1, sCTLA-4, sCD200/sCD200R), generated by alternative splicing or proteolytic cleavage of membrane domains, also play a significant role. Acting as ligand scavengers, they fine-tune checkpoint signaling. Depending on the context, they can limit excessive activation or destabilize the regulatory balance. Their serum concentrations reflect the global regulatory tone and, in glomerulopathies, may act as biomarkers of disease activity and progression of kidney damage [42,43,44,45,46,47,48,49,50,51,52]. In this paper, we aimed to systematically compare the profile of selected immune checkpoints in two key GN—IgAN and MPGN—in relation to each other and to healthy volunteers serving as controls. We analyzed: (1) the percentages of cells expressing PD-1, PD-L1, CTLA-4, CD86, and CD200/CD200R in the CD4+ and CD8+ T cell subpopulations, CD19+ B cells, and CD3-CD16+CD56+ NK cells; (2) serum concentrations of soluble forms of selected molecules; and (3) expression levels of relevant transcripts in PBMC. Additionally, across the entire range of analyses, we assessed whether there were any differences and/or similarities related to patient gender. This multi-level and comparative approach aims to identify common and specific immunoregulatory signatures for IgAN and MPGN, better understand their pathophysiology, and indicate potential biomarkers and targets for future immunomodulatory strategies.

## 2. Materials and Methods

### 2.1. Characteristics of Study Participants, Eligibility Criteria, and Study Material

A total of 90 individuals were enrolled in the project, comprising 60 patients diagnosed with GN and 30 healthy volunteers (HV) who served as controls. Based on histopathological analysis of renal biopsies, two equal subgroups were identified among the GN patients: 30 with IgAN and 30 with MPGN.

The demographic composition of the GN patients included 26 women (43%) and 34 men (57%). The control group consisted of 13 women and 17 men, selected to be age-matched and free from chronic systemic diseases. Inclusion criteria included: age ≥ 18 years, a recent diagnosis of primary GN confirmed by renal biopsy, no prior immunosuppressive therapy or steroid therapy, no active infection at the time of sample collection, and informed consent. Individuals with secondary forms of glomerulopathy (e.g., lupus nephropathy, amyloidosis), HIV, HBV, or HCV infections, autoimmune diseases involving multiple systems, post-organ transplantation, diagnosed hematological or neoplastic diseases, and patients who had received immunomodulatory drugs or antibiotic therapy within the previous three months were excluded.

The study material consisted of peripheral blood collected from the antecubital vein. From each individual, 10 mL of blood was collected in EDTA tubes (intended for PBMC isolation and flow cytometry analysis), and 5 mL was collected in clot tubes to obtain serum for serological testing. Samples were processed immediately after collection, using procedures that ensured cell integrity and protein stability. All procedures were conducted in accordance with the principles of good laboratory and bioethical practice, as approved by the relevant bioethics committee.

### 2.2. Isolation of Peripheral Blood Mononuclear Cells (PBMC)

Peripheral blood mononuclear cells (PBMC) were obtained from whole blood collected in EDTA-containing tubes. A gradient separation method was used for isolation using Gradisol L (Aqua-Med, Łódź,Poland) with a density of 1.077 g/mL, according to the manufacturer’s procedure and descriptions available in the scientific literature. Blood samples were diluted 1:1 with PBS buffer, free of calcium and magnesium ions, and gently transferred onto a layer of separation medium in 15 mL tubes. Centrifugation was then performed at 700× *g* for 20 min at room temperature, without using the rotor brake. After centrifugation, the PBMC-rich layer was collected using a sterile pipette and washed twice in PBS (10 min, 400× *g*, 4 °C) to remove residual gradient solution and cellular components. After the second wash, the resulting cell pellet was resuspended in an appropriate amount of lysis buffer, preparing the material for further RNA isolation. Finally, the PBMC were used for total ribonucleic acid (RNA) extraction using a spin column kit according to the manufacturer’s instructions. The resulting RNA was then used to determine the expression of *PD-1, PD-L1, CTLA-4, CD86, CD200*, and *CD200R* genes using quantitative real-time PCR (qPCR).

### 2.3. RNA Isolation, cDNA Synthesis, and qPCR Analysis

Total RNA was isolated from peripheral blood mononuclear cells (PBMCs) using the Total RNA Mini Plus kit (A&A Biotechnology, Gdansk, Poland) according to the manufacturer’s instructions. Cells were lysed in a buffer containing guanidine and RNase inhibitors and then purified on silica columns. After centrifugation and washing with buffers RW and RPE, ribonucleic acid was eluted in 30 μL of RNase-free water. RNA quantity and purity were assessed spectrophotometrically using a NanoDrop (Shimadzu, Kyoto, Japan), with an A260/A280 ratio greater than 1.8 considered the criterion for sample eligibility for further analysis.

Complementary DNA (cDNA) synthesis was performed using 500 ng of RNA using the iScript™ cDNA Synthesis Kit (Bio-Rad Laboratories, Hercules, CA, USA). The total reaction volume was 20 μL, and the procedure was performed in a thermal cycler according to the following schedule: 5 min at 25 °C (preparation), 30 min at 42 °C (synthesis), and 5 min at 85 °C (enzyme inactivation). The resulting products were stored at −80 °C until further analysis.

The expression of PD-1, PD-L1, CTLA-4, CD86, CD200, and CD200R genes was determined by quantitative real-time PCR (qPCR) using SYBR Green dye. SsoAdvanced™ Universal SYBR^®^ Green Supermix (Bio-Rad, Hercules, CA, USA) was used for amplification, along with specific primers provided by the manufacturer. Each reaction (20 µL) contained: 10 µL Supermix, 1 µL primer, 2 µL cDNA template, and 7 µL nuclease-free water. The amplification program included an initial denaturation step (95 °C, 30 s), followed by 40 cycles (95 °C, 5 s; 60 °C, 30 s), run on a CFX96 Real-Time PCR Detection System (Bio-Rad, Hercules, CA, USA). After amplification, melting curve analysis was performed to confirm the specificity of the products. Gene expression was normalized to the reference gene β-actin, and the data were interpreted using relative expression analysis (2^−ΔΔCt^).

### 2.4. Immunophenotyping and Analysis of PD-1, PD-L1, CTLA-4, CD86, CD200 and CD200R Expression on Lymphocyte Subpopulations

The phenotypic profile of peripheral blood lymphocytes was assessed by multiparameter flow cytometry. The following monoclonal antibodies were used to label surface antigens: anti-CD3 BV510 (clone HIT3a, cat. 564713, BD Biosciences, NY, USA), anti-CD4 APC-R700 (clone OKT4, cat. 566808, BD Biosciences), anti-CD8 BV605 (clone HIT8a, cat. 569169, BD Biosciences), anti-CD16 BV650 (clone 3G8, cat. 563692, BD Biosciences), anti-CD19 PerCP (clone SJ25C1, cat. 332780, BD Biosciences), and anti-CD56 BV650 (clone NCAM16.2, cat. 564057, BD Biosciences). For the regulatory molecules PD-1, PD-L1, CTLA-4, CD86, CD200, and CD200R, the following antibodies were used: anti-PD-1 PE (clone MIH4, cat. 557946, BD Biosciences), anti-CD200 PE (clone MRC OX-104, cat. 552475, BD Biosciences), anti-CTLA-4 PE (clone BNI3, cat. 560939, BD Biosciences), anti-PD-L1 APC (clone MIH1, cat. 563741, BD Biosciences), anti-CD200R APC (clone OX-108, cat. 329308, BioLegend, San Diego, CA, USA), and anti-CD86 APC (clone BU63, cat. 571636, BD Biosciences). To reduce interference from fluorescent signal overlap, BD Horizon™ Brilliant Stain Buffer Plus (BD Biosciences, cat. 566385) was added to the mixture. Samples were then incubated in the dark at room temperature for 20 min and then washed in BD Pharmingen™ Stain Buffer (FBS) (BD Biosciences, cat. 554656). For intracellular staining, the BD Cytofix/Cytoperm™ Fixation/Permeabilization Kit (BD Biosciences, cat. 554714) was used according to the manufacturer’s instructions. Measurements were performed using a CytoFLEX LX cytometer (Beckman Coulter, Brea, CA, USA), and data were analyzed using Kaluza v2.1 software (Beckman Coulter). Fluorescence signal compensation was performed using the VersaComp Antibody Capture Kit (Beckman Coulter, cat. B22804). Instrument performance was monitored daily using CytoFLEX Daily QC Fluorospheres (Beckman Coulter, cat. C65719). Fluorescence minus one (FMO) controls were prepared to ensure the accuracy of the gating strategy. The detailed gating scheme is presented in Figure S1 in the Supplementary Materials.

### 2.5. Determination of Serum Control Point Concentrations by ELISA

Soluble levels of the regulatory proteins PD-1, PD-L1, CTLA-4, CD86, CD200, and CD200R were determined by sandwich enzyme-linked immunosorbent assay (ELISA) using commercial kits from Wuhan Fine Biotech Co., Ltd. (FineTest, Wuhan, China). All steps of the procedure were performed according to the manufacturer’s protocols. Absorbance was measured at 450 nm using a Victor^3^ microplate reader (PerkinElmer, Waltham, MA, USA). The obtained values were converted to concentrations expressed in pg/mL or ng/mL according to the specifications of the individual diagnostic kits.

### 2.6. Statistical Analysis

Results were analyzed using GraphPad Prism v.9.0 software (GraphPad Software Inc., San Diego, CA, USA). The Shapiro–Wilk test was used to assess the normality of the distribution of the studied variables. Because most of the analyzed parameters did not meet the criteria for normal distribution, nonparametric statistical methods were employed.

The Mann–Whitney U test was used for comparisons between two independent groups, while the Kruskal–Wallis test was used for multigroup analyses (including subgroups of patients with GN: IgAN and MPGN, and the control group). When the overall analysis revealed statistical significance (*p* < 0.05), Dunn’s post hoc test was used to identify differences between individual pairs of groups.

A *p* < 0.05 was considered the level of statistical significance. Data were presented as medians and quartiles Q1 and Q3. To assess the diagnostic value of the studied parameters—such as TLR expression and serum checkpoint concentrations—ROC (Receiver Operating Characteristic) curve analysis was performed. Based on the obtained curves, the area under the curve (AUC) was calculated as an indicator of the parameter’s ability to differentiate patients with GN from healthy individuals and to distinguish between subtypes of glomerulopathy. The 95% confidence interval (CI) for the AUC and the corresponding *p* value were also determined for each variable.

## 3. Results

### 3.1. Analysis of Basic Hematological, Biochemical, and Immunological Parameters of Patients with IgAN, MPGN, and Healthy Volunteers Recruited to the Study

We enrolled 60 patients with GN—30 with IgAN and 30 with MPGN—and 30 age-matched healthy volunteers (HVs) as controls, applying the inclusion/exclusion criteria detailed in Materials and Methods (Table 1). Sex distribution was similar in IgAN and HVs (56.7% men; 43.3% women), whereas MPGN had a higher male proportion (66.7% men; 33.3% women). The groups were comparable in age: median (Q1–Q3) was 43.0 (37.25–51.0) years in IgAN, 48.5 (39.5–54.0) in MPGN, and 44.5 (35.0–70.0) in HV; no statistically significant differences were found either overall (*p* = 0.5533) or in pairwise comparisons (all *p* > 0.9999). Initially, basic laboratory parameters were assessed (Table 1). Compared to the control group, patients had higher total leukocyte counts, and additionally, MPGN had higher neutrophil counts than IgAN, with no significant differences in the percentages of monocytes and lymphocytes. Eosinophils and basophils were also elevated in MPGN compared to controls. Erythropoietic profiles indicated that IgAN patients had lower erythrocyte and hemoglobin values than MPGN patients and healthy controls, consistent with renal anemia. Platelet counts did not differ significantly between groups. Renal function assessment revealed greater impairment in IgAN (higher creatinine and urea, and lower eGFR) compared to HV. At the same time, MPGN had the highest levels of proteinuria, suggesting more severe impairment of the filtration barrier. In terms of lipid metabolism, patients with MPGN exhibited an atherogenic profile compared to controls, characterized by higher concentrations of total cholesterol, triglycerides, and LDL, and lower HDL levels. Among immunoglobulins, higher concentrations of IgA and IgG were observed in IgAN than in MPGN and in the HV, consistent with the pathogenic mechanisms of this disease. In both groups, albumin and total protein concentrations were reduced compared to controls, consistent with the concomitant proteinuria (Table 1).

In the analysis of lymphocyte subpopulations, no significant differences were found between patients with IgAN and MPGN and the control group (Table 2). The percentage of CD45+ cells was high and comparable in all study groups, indicating preserved integrity of the leukocyte population. Similarly, the distribution of total T lymphocytes (CD3+), helper T lymphocytes (CD4+), and cytotoxic T lymphocytes (CD8+) remained similar regardless of diagnosis. The CD4+/CD8+ ratios were within normal limits and showed no statistically significant differences. The percentage of B lymphocytes (CD19+) and NK cells (CD3-CD16+CD56+) also did not differ significantly between groups (Table 2).

### 3.2. Expression of Immune Checkpoints on the Cell Surface, Concentrations of Their Serum Soluble Forms, and Transcript Levels in PBMCs from Patients with IgAN, MPGN, and Controls

**Figure 1:**
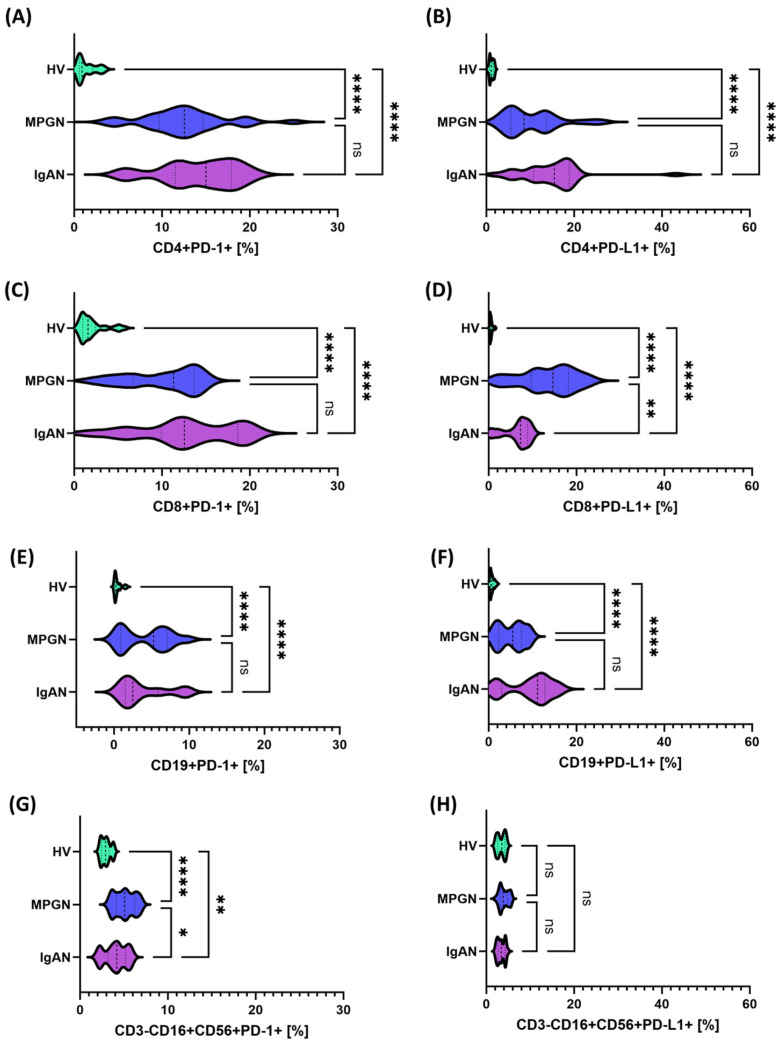
Expression of PD-1 and PD-L1 checkpoints on selected immune cell subpopulations in patients with IgAN (purple), MPGN (blue), and healthy volunteers (HV, green). (**A**) CD4+PD-1+, (**B**) CD4+PD-L1+, (**C**) CD8+PD-1+, (**D**) CD8+PD-L1+, (**E**) CD19+PD-1+, (**F**) CD19+PD-L1+, (**G**) CD3-CD16+CD56+PD-L1+, (**H**) CD3-CD16+CD56+PD-1+. Data are presented as violin plots with median and quartiles. Significant statistical differences are indicated by: * *p* < 0.05, ** *p* < 0.001, ****p < 0.0001, ns—not significant. Further analysis assessed the expression of molecules from the CTLA-4/CD86 axis in comparison between patients with GN and HV. In contrast to the PD-1/PD-L1 pathway, an opposite trend was observed in this case—the percentage of cells expressing CTLA-4 and CD86 in the analyzed subpopulations was statistically significantly lower in patients with GN compared to HV (Figure 2). Comparative analysis between GN subunits also showed that significant differences were observed only in the NK cell population (CD3-CD16+CD56+CTLA-4+), where the values were higher in patients with IgAN than in those with MPGN (Figure 2G).

To precisely assess differences in immune response regulation between patients with IgAN, MPGN, and HV, we analyzed the expression of selected immune checkpoints and their ligands (PD-1/PD-L1, CTLA-4/CD86, and CD200/CD200R) both on the surface of immune cell subpopulations, in soluble forms, and at the transcriptomic level (Supplementary Materials Table S1). In the analysis of the PD-1/PD-L1 pathway, a statistically significant increase in the percentage of cells expressing these molecules was observed in all studied subpopulations of T, Blymphocytes and NK cells in patients with GN compared to HV. The only exception was the lack of significant differences in PD-L1 expression on NK cells (Figure 1). Comparative analysis between GN subunits revealed statistically significant differences only in CD8+PD-L1+ lymphocytes and CD3-CD16+CD56+PD-1, with higher expression observed in MPGN patients compared to IgAN patients in both cases (Figure 1D,G).

**Figure 3:**
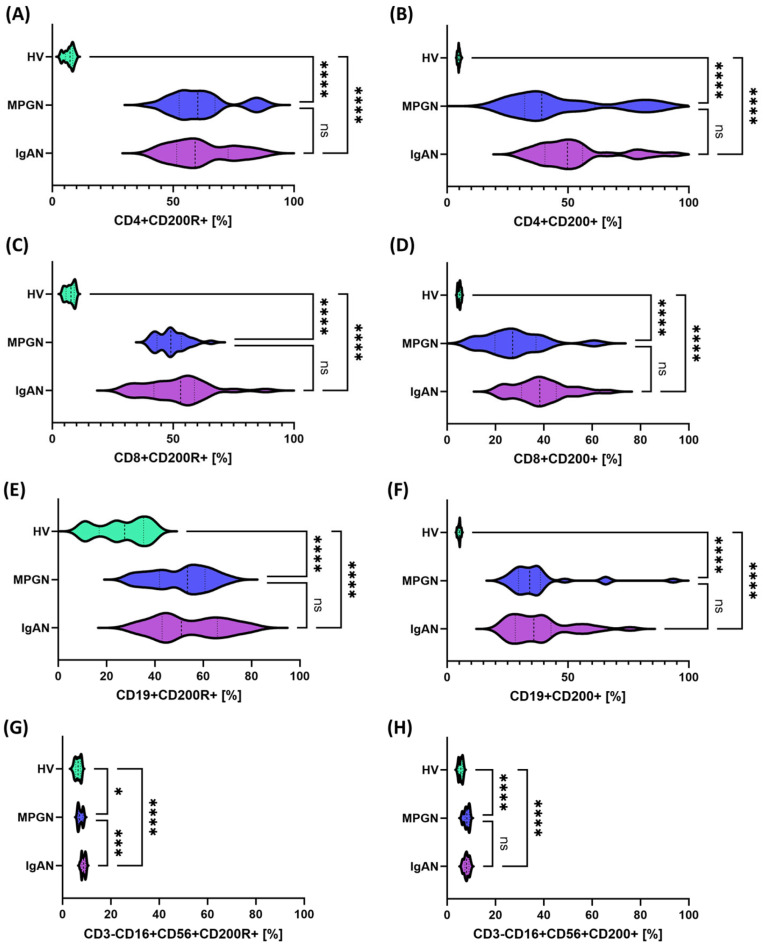
Expression of CD200 and CD200R molecules on the surface of selected immune cell subpopulations in patients with IgAN (purple), MPGN (blue), and healthy volunteers (HV, green). (**A**) CD4+CD200R+, (**B**) CD4+CD200+, (**C**) CD8+CD200R+, (**D**) CD8+CD200+, (**E**) CD19+CD200R+, (**F**) CD19+CD200+, (**G**) CD3-CD16^+^CD56+CD200R+, (**H**) CD3-CD16+CD56+CD200+. Data were presented as violin plots, taking into account the median, quartiles, and distribution of values in the study groups. Statistical differences between groups were marked: * *p* < 0.05, *** *p* < 0.001, **** *p* < 0.0001, ns—not significant.

Analysis of the expression of the last examined CD200R/CD200 pathway revealed a significant increase in the percentage of positive cells across all assessed lymphocyte subpopulations and NK cells in patients with GN compared to HV (Figure 3). Comparison between the individual GN subunits revealed that statistically significant differences occurred only in CD3-CD16+CD56^+^CD200R+, where the values were higher in patients with IgAN than in those with MPGN (Figure 3G).

### 3.3. Comparison of Serum Concentrations and Gene Expression of Selected Immune Checkpoints and Their Ligands in Patients with IgAN, MPGN, and HV

**Figure 4:**
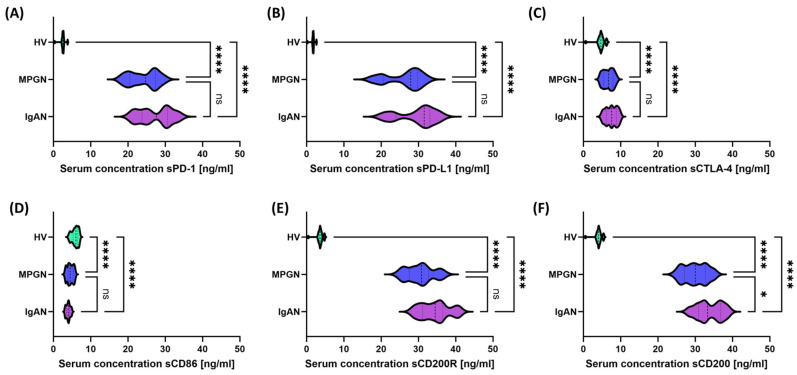
Serum concentrations of soluble immune checkpoints in patients with IgAN (purple), MPGN (blue), and healthy volunteers (HV, green). (**A**) sPD-1, (**B**) sPD-L1, (**C**) sCTLA-4, (**D**) sCD86, (**E**) sCD200R, (**F**) sCD200. Data are presented as violin plots, illustrating the median, quartiles, and full distribution of values across study groups. Significant differences between groups are indicated by: * *p* < 0.05, **** *p* < 0.0001, ns—not significant. Analysis of checkpoint gene expression in PBMCs revealed significant differences between IgAN, MPGN, and HV (Figure 5). *PD-1* (Figure 5A) was higher in MPGN compared to HV, and in IgAN it had intermediate values, also differing from HV; no differences were found between IgAN and MPGN. For *PD-L1* (Figure 5B), a similar trend was observed, with a predominance of MPGN, but without significant changes between IgAN and HV or between the subgroups themselves. Expression of *CTLA-4* (Figure 5C) and *CD86* (Figure 5D) was higher in GN than in HV, but without significant differences between HV; significant differences were observed between IgAN and MPGN. *CD200R* and *CD200* (Figure 5E,F) exhibited a pattern similar to that of PD-1/PD-L1 in comparisons with HV, with no significant differences observed between IgAN and MPGN.

A consistent pattern of checkpoint dysregulation was observed in the serum of patients with GN: increased sPD-1 and sPD-L1 (Figure 4A,B) and sCD200R and sCD200 (Figure 4E,F) with concomitant decreased sCTLA-4 and sCD86 (Figure 4C,D) relative to HV. Comparison of IgAN with MPGN revealed no differences for most molecules, except for higher sCD200 in IgAN (Figure 4F). The pattern of these changes suggests a global disruption of the mechanisms that inhibit the immune response in GN.

### 3.4. Similarities and Differences in the Assessment of Immunological Control Parameters Depending on the Gender of Patients with IgAN, MPGN, and HV

Biological sex may modulate lymphocyte activity, cytokine profile, and checkpoint expression, therefore we assessed sex-related differences in IgAN, MPGN, and HV. We first verified standard hematological and biochemical parameters (Supplementary Materials, Table S2) and then analyzed immune checkpoints. The most pronounced differences were observed in comparisons of GN patients (female and male) to their HV counterparts; additionally, higher basophils were observed in IgAN Male vs. MPGN Male, higher IgG in IgAN Male vs. MPGN Male and IgAN Female vs. MPGN Female, and higher total protein in IgAN Female vs. MPGN Female (Supplementary Materials, Table S3). In the PD-1/PD-L1 axis, most significant differences were observed in GN vs. HV (Supplementary Material, Figure S2), with no changes for CD3-CD16+CD56+PD-L1 (Figure S2H). the only differences between subtypes and gender were CD3-CD16+CD56+PD-1+ (IgAN Female vs. MPGN Male and IgAN Female vs. MPGN Female; higher in MPGN, Figure S2G). For the CTLA-4/CD86 pathway, the differences were weaker both in GN vs. HV and between IgAN and MPGN; the only significant signal was the percentage of CTLA-4 on NK cells in IgAN Males vs. MPGN Females (Supplementary Material, Figure S3G). A similar picture was obtained for CD200R/CD200 vs. PD-1/PD-L1 (Figure 4): most differences were in GN vs. HV, and the only difference between subtypes was CD3-CD16+CD56+CD200R+ in IgAN Male vs. MPGN Male (Supplementary Material, Figure S4). Global checkpoint deregulation was confirmed in serum (elevated selected soluble forms; Supplementary Material, Figure S5), but gender analysis did not reveal a significant effect on its severity. Gene expression in PBMC indicates that the deregulation profile depends primarily on the disease type (IgAN vs. MPGN) and not on gender, with no significant differences between subgroups or with respect to HV (Supplementary Material, Figure S6).

### 3.5. Common Biochemical-Immunological Correlations in IgAN and MPGN as a Potential Reflection of Similar Pathogenetic Mechanisms

To more comprehensively assess the associations between immunological and clinical parameters in the IgAN and MPGN patient groups, Spearman’s rank correlation analysis was performed. In the case of IgAN, a total of 132 statistically significant correlations were identified, comprising 62 negative correlations (22 weak, 37 moderate, and 3 strong) and 69 positive correlations (30 weak, 30 moderate, 6 strong, and 3 very strong). In the MPGN group, 139 significant correlations were identified, including 53 negative (23 weak, 30 moderate) and 85 positive (26 weak, 53 moderate, and 6 strong) (Supplementary Materials Tables S4 and S5). Comparative analysis also revealed a set of common correlations observed simultaneously in both diseases. Consistent, positive associations were observed between biochemical parameters in both diseases (Table 3). Albumin strongly correlated with IgG and total protein in both MPGN (R = 0.509–0.603) and IgAN (R = 0.673–0.718), as well as with urea; however, this association was weaker in IgAN (R = 0.384 vs. 0.566). Similarly strong correlations were observed between creatinine and urea (MPGN: R = 0.510; IgAN: R = 0.871) and between creatinine and uric acid (R = 0.413 vs. 0.609), suggesting metabolic connections characteristic of renal failure. In terms of lipids, both groups exhibited a positive correlation between cholesterol and LDL (MPGN: R = 0.534; IgAN: R = 0.839) and proteinuria (R = 0.459 vs. 0.379), suggesting a role for lipid abnormalities in the progression of glomerular damage. Regarding immunoglobulins, the correlation between IgG and total protein was stronger in IgAN (R = 0.793 vs. 0.638).

Immune checkpoint analysis revealed significant differences in the direction of correlation. Negative correlations were more frequently observed in MPGN, for example, between CD19+CD86+ and CD19+PD-L1+ (R = –0.562), or between CD3–CD16+CD56+CTLA-4+ and CD4+CD86+ (R = –0.546), which may suggest dysregulation of suppressive mechanisms. In IgAN, these same axes were positive (R = 0.370 and 0.430). A similar contrast was observed between CD4+CD200R+ and CD8+PD-1+ (R = 0.485 in MPGN vs. R = –0.539 in IgAN), indicating differences in the way inhibitory receptors are engaged in both diseases. However, the relationships between soluble forms were stable: in both groups, there was a positive correlation between sPD-1 and sPD-L1 (MPGN: R = 0.380; IgAN: R = 0.375), as well as between qCD200R and qPD-1 and qCD86 and qPD-L1, reflecting a typical pattern of dysregulation in GN.

### 3.6. ROC-Based Assessment of Similarities and Differences in Immune Checkpoint Profiles Between IgAN, MPGN, and Healthy Controls

Evaluating the diagnostic and differential value of immunological biomarkers is a crucial aspect of research into the pathogenesis of GN. Receiver Operating Characteristic (ROC) curve analysis enables the determination of the sensitivity and specificity of the studied parameters, as well as their ability to differentiate between individual disease entities and healthy volunteers. In the context of the immune system, immune checkpoints such as PD-1/PD-L1, CTLA-4/CD86, and CD200/CD200R are of particular interest, as their dysregulation may play a key role in maintaining chronic lymphocyte activation and disrupting the balance between tolerance and inflammatory response. In this analysis, we used ROC curves to assess the potential of individual checkpoints as markers differentiating patients with IgAN and MPGN from HV, as well as to compare these two disease entities. The approach used not only allows for the identification of parameters of the highest diagnostic value but also indicates potential differences in immunoregulatory mechanisms between IgAN and MPGN. Detailed data are presented in Supplementary Materials Table S6.

**Figure 6:**
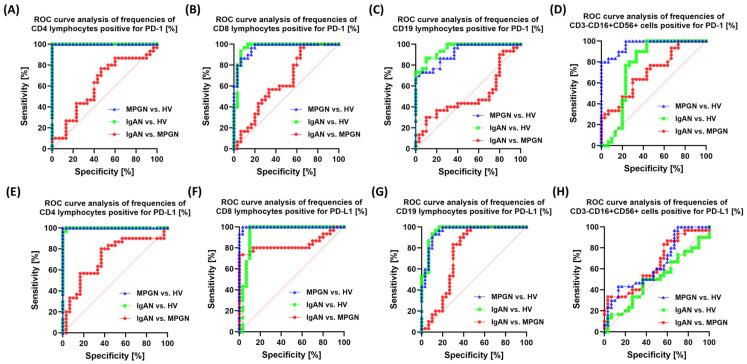
Analysis of ROC curves for the percentage of PD-1 and PD-L1-positive cells in selected immune subpopulations in patients with IgAN, MPGN, and HV. (**A**) CD4+PD-1+, (**B**) CD4+PD-L1+, (**C**) CD8+PD-1+, (**D**) CD8+PD-L1+, (**E**) CD19+PD-1+, (**F**) CD19+PD-L1+, (**G**) CD3-CD16+CD56+PD-L1+, (**H**) CD3-CD16+CD56+PD-1+. The graphs present ROC curves illustrating the ability of individual parameters to differentiate patients with IgAN and MPGN from the control group (HV), as well as to distinguish between the two diseases. The y- and x-axis values correspond to sensitivity (%) and specificity (%), respectively.

ROC curve analysis showed that the percentages of PD-1 and PD-L1-positive cells in selected lymphocyte and NK cell subpopulations were highly capable of differentiating patients with glomerulopathies (IgAN, MPGN) from HV. The highest diagnostic power was observed for CD4+PD-1+ and CD4+PD-L1+ lymphocytes, which were particularly effective in distinguishing patients with GN from HV (Figure 6). Analysis of the differences between IgAN and MPGN revealed that some parameters, especially in the CD8+ T lymphocyte subpopulations, demonstrated moderate ability to distinguish between the two disease entities.

**Figure 7:**
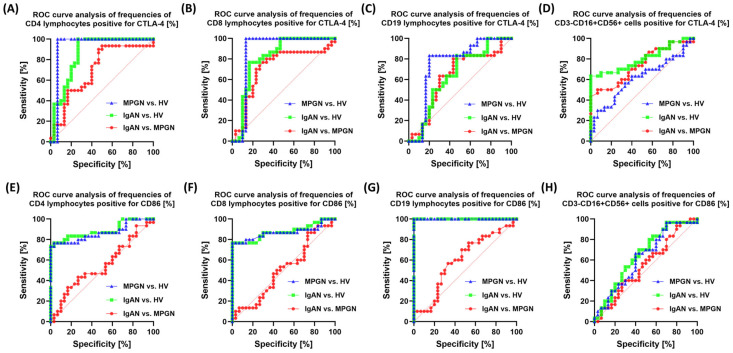
ROC curve analysis for the percentage of CTLA-4 and CD86-positive cells in selected immune subpopulations in patients with IgAN, MPGN, and HV. (**A**) CD4+CTLA-4+, (**B**) CD4+CD86+, (**C**) CD8+CTLA-4+, (**D**) CD8+CD86+, (**E**) CD19+CTLA-4+, (**F**) CD19+CD86+, (**G**) CD3-CD16+CD56+CTLA-4+, (**H**) CD3-CD16+CD56+CD86+. The graphs present ROC curves that define the ability of the tested parameters to differentiate patients with IgAN and MPGN from HV and between the two disease entities.

In the analysis of the sensitivity and specificity of the percentage of cells expressing CTLA-4 and CD86, the highest diagnostic value was obtained for the CD19+CTLA-4+ population in comparison to patients with GN and HV (Figure 7). In differentiating between IgAN and MPGN, only moderate sensitivity and specificity were observed for the analyzed molecules, indicating their limited usefulness in distinguishing between these two entities.

**Figure 8:**
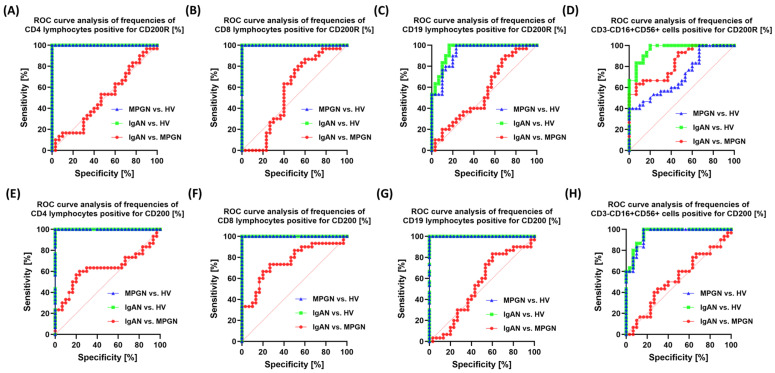
ROC curve analysis for the percentage of CD200R and CD200-positive cells in selected immune subpopulations in patients with IgAN, MPGN, and HV. (**A**) CD4+CD200R+, (**B**) CD4+CD200+, (**C**) CD8+CD200R+, (**D**) CD8+CD200+, (**E**) CD19+CD200R+, (**F**) CD19+CD200+, (**G**) CD3-CD16+CD56+CD200R+, (**H**) CD3-CD16+CD56+CD200+. The graphs show ROC curves illustrating the ability of individual parameters to differentiate patients with IgAN and MPGN from the control group (HV) and to distinguish between the two diseases. The values on the axes correspond to sensitivity (%) and specificity (%), respectively.

Analysis of the latter pathway revealed that the highest sensitivity and specificity were observed for the percentage of CD200R on CD4+ and CD8+ cells, as well as CD200 on CD4+, CD8+, and CD19+ cells, among patients with IgAN, MPGN, and HV (Figure 8). The sensitivity and effectiveness of these molecules for differentiating between the two subgroups were, as in the previous cases, quite limited.

**Figure 9:**
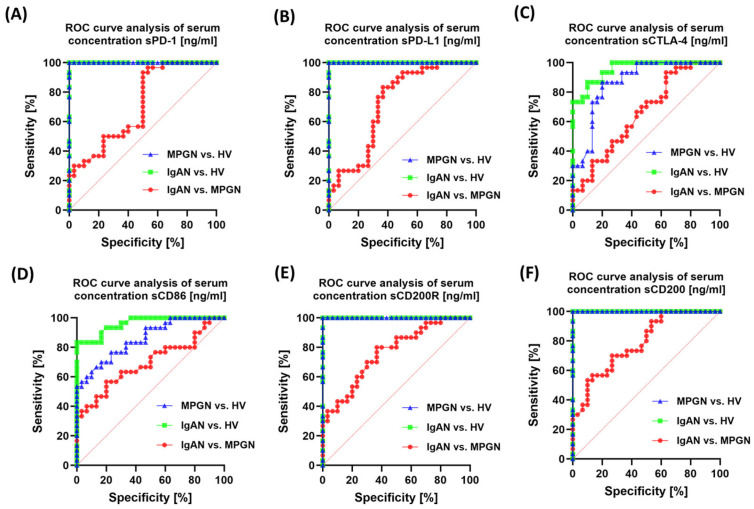
Analysis of ROC curves for serum concentrations of soluble forms of immune checkpoints in patients with IgAN, MPGN, and HV. (**A**) sPD-1, (**B**) sPD-L1, (**C**) sCTLA-4, (**D**) sCD86, (**E**) sCD200R, (**F**) sCD200. The graphs present ROC curves defining the sensitivity (%) and specificity (%) of individual parameters in differentiating patients with IgAN and MPGN from HV, as well as in distinguishing both disease subunits from each other.

**Figure 10:**
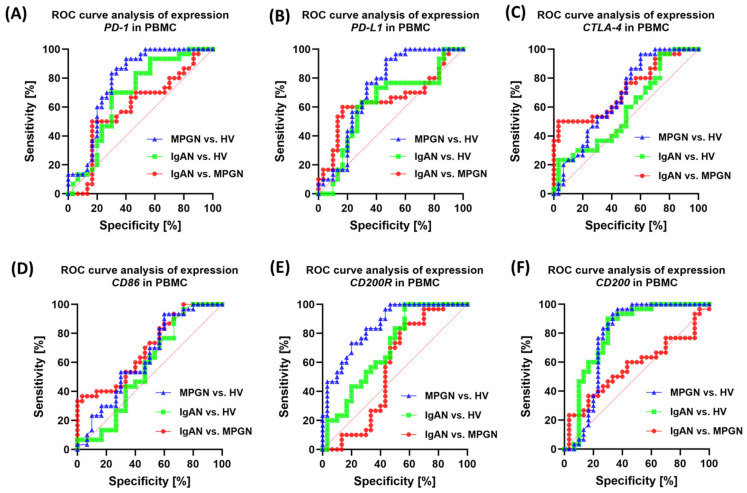
ROC curve analysis for the expression of genes encoding immune checkpoints in peripheral blood mononuclear cells (PBMC) in patients with IgAN, MPGN, and HV. (**A**) PD-1, (**B**) PD-L1, (**C**) CTLA-4, (**D**) CD86, (**E**) CD200R, (**F**) CD200. The graphs present ROC curves illustrating the sensitivity (%) and specificity (%) of the tested parameters in differentiating patients with IgAN and MPGN from the control group (HV), as well as in distinguishing between the two subunits of GN.

When assessing the sensitivity and specificity of the soluble test molecules in the serum, the highest values were observed for sPD-1 and sPDL-1, as well as sCD200R and sCD200, in IgAN, MPGN, and HV (Figure 9). Moderate values for the tested molecules were observed in differentiating the individual subunits. The assessment of the expression of the tested immune checkpoints in PBMC proved to be significantly less effective and sensitive, demonstrating moderate sensitivity in the evaluation of IgAN, MPGN, and HV, as well as between the subunits (Figure 10).

## 4. Discussion

### 4.1. The Role of the PD-1/PD-L1, CTLA-4/CD86 and CD200/CD200R Axes in the Pathogenesis of IgAN and MPGN

Glomerulonephritis, such as IgA nephropathy (IgAN) and membranoproliferative glomerulonephritis (MPGN), is a disease entity that develops at the intersection of chronic immune activation, genetic predisposition, and environmental influences. A crucial element of this balance is the immune checkpoint, which modulates the activation and inhibition of lymphocytes and innate cells. Available literature suggests that checkpoints such as PD-1, PD-L1, CTLA-4, and CD86 play a significant role in regulating inflammatory responses in the kidneys. Although conclusive clinical studies are lacking in IgA nephropathy and MPGN, increasing evidence is emerging from analyses conducted in other glomerulopathies, such as lupus nephritis, which serves as a model for autoimmune kidney disease in many respects. In this disease, an increase in PD-1 and PD-L1 expression is observed, which correlates with disease activity. Additionally, modulation of this pathway in experimental models led to a reduction in glomerular damage [53,54,55].

One of the goals of our study was to simultaneously assess all pathways in the study patient, aiming to identify a consistent profile of these pathways in patients compared to healthy volunteers. Our analyses showed that at the cellular level, using flow cytometry, we observed a higher percentage of cells expressing PD-1/PD-L1+ in T (CD4+, CD8+) and B (CD19+) lymphocytes, as well as an increase in PD-1 on NK cells, with no differences in PD-L1 expression in this population in patients compared to healthy volunteers. At the same time, we observed increased levels of soluble forms of sPD-1 and sPD-L1 (Figure 4), which could suggest activation of the PD-1/PD-L1 inhibitory axis and/or partial “exhaustion” of chronically stimulated lymphocytes. Our analyses align with studies suggesting that in the context of IgA nephropathy, PD-1 is often overexpressed on T lymphocytes, potentially leading to a dysregulated immune response that contributes to disease pathogenesis [56]. Moreover, there are also reports suggesting that by inhibiting T cell activation, PD-1 may allow IgA deposits to persist in the kidneys, potentially leading to inflammation and kidney damage. At the same time, the use of PD-1/PD-L1 interaction blockade has proven to be a justification for therapeutic interventions aimed at restoring T lymphocyte activity and preventing the abnormal accumulation of IgA [57]. Other studies related to IgA nephropathy and PD-1 coenzyme also indicated that patients with IgAN have an increased percentage of T peripheral helper (Tph) cells expressing the PD-1 population and CD138+ B cells. These investigators reported that the percentage of these cells correlated positively with proteinuria and negatively with glomerular filtration rate. With the use of immunosuppressive treatment, the percentage of Tph and CD138+ B cells, as well as IL-21 levels, decreased, concomitant with an improvement in glomerular filtration rate [58].

A different trend was observed for molecules belonging to the CTLA-4/CD86 axis. In this respect, the studied lymphocyte subpopulations showed a statistically significant decrease in the percentage of CTLA-4+ and CD86+ cells, and decreased serum concentrations of sCTLA-4 and sCD86 were observed, suggesting a weakening of CTLA-4/CD86-dependent inhibition/co-stimulation in the circulation. At the same time, in the context of increased expression of PD-1/PD-L1, CD200/CD200R, and decreased expression of CTLA-4/CD86, it can be assumed that the body responds by activating checkpoints as a result of chronic immune stimulation. Differences in CTLA-4 and CD86 levels are observed not only in our studies. Studies have shown that the balance between activating and inhibitory signals of T lymphocytes may be a key element in susceptibility to IgA nephropathy [59]. At the same time, CTLA-4 and CD86 polymorphisms are associated with the risk of kidney transplant rejection [60]. This confirms the importance of these checkpoints, and at the same time, the role of CTLA-4, present on regulatory lymphocytes, seems to be crucial, as it limits their availability by interacting with CD80/CD86, thereby increasing the pool of free PD-L1 and promoting the inhibition of the immune response [61]. In turn, when it comes to the role of CD200 and CD200R molecules, there is a significant gap in research and available literature. Typically, these molecules are analyzed in oncology and autoimmune disease studies and are considered potentially important checkpoints. However, in the context of glomerulonephritis, data are practically nonexistent [62]. To our knowledge, our analyses provide some of the first data on this topic. Furthermore, in our further analyses, targeting CD200 and CD200R molecules, we observed increased expression in all analyzed subpopulations (T, B, NK) and in serum (sCD200, sCD200R), which may suggest that the CD200/CD200R axis is involved in a feedback mechanism aimed at limiting inflammation. Our analyses revealed that patients with glomerulopathy (both IgAN and MPGN) exhibit significantly higher expression of CD200 and CD200R on T lymphocytes (CD4+ and CD8+), B lymphocytes, and NK cells compared to healthy controls. Furthermore, serum concentrations of soluble CD200 and sCD200R were elevated in patients. These results seem to contradict the PD-1/CTLA-4 changes discussed above, as they indicate increased activation of inhibitory mechanisms. However, in our opinion, we propose that their interpretation be a compensatory response of the immune system to chronic inflammation in the kidneys, which is triggered by signals from, among others, the kidneys. In other words, the increase in CD200/CD200R may be a consequence of prolonged activation, with the body activating every available suppressive pathway.

Regarding the data, however, a comparative analysis between IgAN and MPGN revealed narrow and quantitative differences that do not alter the fundamental picture of the common disorders. In MPGN, CD8+PD-L1+ and CD3-CD56+CD16+PD-1+ were more frequent, which may indicate more potent inhibition of the CD8/NK axis under conditions of intense stimulation. In IgAN, we observed a relatively higher CD200R on NK and a higher concentration of sCD200, as well as higher CTLA-4 expression on NK compared to MPGN, suggesting a greater contribution of the CD200-CD200R regulatory mechanisms and the CTLA-4-dependent component in modulating the innate immune response. Stratified analysis confirms this interpretation: the protein phenotype on the cell surface and serum profiles were consistent and clearly different from controls, while gene expression in IgAN reflected the changes less strongly—for PD-1/PD-L1, a trend of increase was visible mainly in MPGN compared to healthy controls, and for CTLA-4/CD86, no significance was observed vs. HV, with simultaneous differences between IgAN and MPGN. This discrepancy between the mRNA and protein/serum layers is consistent with the dominant role of non-transcriptional regulation. However, analysis of the gender differences in the study group did not change the main conclusions: the observed effects were primarily related to the comparison of “patients vs. HV” and not “IgAN vs. MPGN” within gender; individual differences in NK subpopulations were point-like.

However, the assessment of clinical utility using ROC curves indicates that the best ability to distinguish patients from healthy controls is possessed by: the percentage of CD4+PD-1+ and CD4+PD-L1+ (surface phenotype), selected indicators of the CD200/CD200R axis (especially CD200 on CD4+/CD8+/CD19+ and CD200R on CD4+/CD8+), and serum sPD-1/sPD-L1/sCD200/sCD200R. In turn, differentiation between IgAN and MPGN was moderate, most pronounced in selected CD8 subpopulations with PD-1/PD-L1. This suggests that a panel combining the PD-1/PD-L1 phenotype (especially in CD4+ cells) with serum PD-1/PD-L1 and CD200/CD200R levels may be helpful in non-invasive assessment of disease activity and monitoring. However, differentiating between IgAN and MPGN would require integrating biomarkers with clinical and histological data. However, it is essential to conduct further studies on a larger group of patients.

It is worth emphasizing that the presented results refer to the immune complex-associated MPGN (IC-MPGN), in which chronic stimulation of the immune response is the main mechanism of the disease. They cannot be directly applied to C3 nephropathy (C3G), which includes C3 glomerulonephritis and dense deposit disease and is characterized by primary dysregulation of the alternative complement pathway rather than a dominant role of immune complexes [63]. In C3G, immune checkpoint dysregulation may be secondary or less significant compared to complement activation mechanisms. Therefore, our observations should be interpreted in the context of IC-MPGN, and further studies are necessary to verify the extent to which analogous abnormalities also occur in C3G, which has a distinct pathogenesis and distinct therapeutic implications.

### 4.2. Therapeutic Potential of Immune Checkpoints in GN

In recent years, increasing attention has been paid to the role of immune checkpoints in the pathogenesis and treatment of kidney diseases. On the one hand, drugs blocking the PD-1/PD-L1 or CTLA-4 receptors, widely used in oncology, have highlighted the importance of these axes through the occurrence of adverse effects in the form of immune-mediated nephropathies, including glomerulonephritis. This phenomenon highlights that disinhibition of regulatory systems promotes the development of chronic inflammation in the glomeruli, and thus suggests that the opposite approach—enhancing inhibitory mechanisms—could have therapeutic potential [64,65,66,67,68,69,70].

However, clinical experience to date is limited. The best-studied example is abatacept (CTLA-4-Ig), whose use in lupus nephropathy did not improve treatment outcomes compared to standard immunosuppression, indicating difficulties in translating the concept of checkpoint enhancement into clinical practice [71,72,73]. Preclinical studies suggest that agonistic modulation of the PD-1/PD-L1 axis, e.g., using a PD-L1-Fc fusion protein, may limit T cell infiltration, reduce cytokine production, and improve renal function parameters in animal models [53]. Similar immunotolerogenic effects are attributed to activation of the CD200R receptor, which inhibits the inflammatory response and promotes the maintenance of tissue tolerance, including in the kidney [74].

Currently, the treatment of immune complex-mediated GN remains based on glucocorticosteroids, cytotoxic drugs, and, in selected cases, rituximab, while complement-targeted therapies are a rapidly developing area [75,76,77,78,79]. In this context, immune checkpoint modulators should be considered a research prospect. Their potential use requires the development of strategies for selectively stimulating inhibitory mechanisms, preferably with limited systemic action, and precise patient selection based on biomarkers such as receptor expression on peripheral blood cells or concentrations of soluble checkpoint forms. Preclinical studies support the concept of using immune checkpoint agonists in immune complex-mediated glomerulopathies, but there is no clear clinical evidence of their effectiveness. It appears that the future of this strategy will depend on a combination of pathogenetic data, biomarkers, and innovative forms of targeted therapy that will effectively and safely restore the balance between activating and inhibiting mechanisms in the immune system.

### 4.3. Limitations of the Study

Several limitations of this study should be acknowledged. First, the sample size, although carefully age- and sex-matched, was relatively small and drawn from a single center, which may restrict the generalizability of the findings to broader populations with IgAN and MPGN. Larger, multicenter cohorts are needed to confirm the reproducibility and robustness of the observed patterns of immune checkpoint dysregulation. Second, the study design was cross-sectional, which limited the ability to determine causality or assess dynamic changes in checkpoint expression and soluble forms over the course of disease progression or treatment. Longitudinal studies would be required to evaluate the prognostic utility of these markers and their value in monitoring therapeutic responses. Third, although a comprehensive, multi-level approach was applied—including cell-surface, soluble, and transcriptomic analyses—the functional consequences of altered checkpoint expression were not directly assessed. Functional assays, such as T-cell exhaustion or cytokine secretion tests, would provide further insight into the biological relevance of these findings. Finally, although gender-related comparisons were included, the study was not powered to detect subtle sex-specific differences. Additionally, demographic or genetic factors (e.g., HLA profiles, complement gene variants) were not considered, which may contribute to inter-individual variability in immune regulation. It is worth emphasizing that the study included only newly diagnosed and immunosuppressed patients, suggesting that the observed changes in immune checkpoints may reflect the natural course of IgAN and MPGN pathogenesis rather than a treatment effect. However, as a cross-sectional study, it does not allow us to determine whether these changes are a primary driver of the disease process or a secondary consequence of chronic immune activation. A limitation of our study is the lack of analysis related to histopathological changes in renal biopsies. Although we performed correlations with clinical parameters (proteinuria, eGFR, hematological, and biochemical parameters), further studies are needed to assess the relationship between control points and the severity of morphological changes.

## 5. Conclusions

This study demonstrates that patients with IgAN and MPGN share a common immunological checkpoint signature characterized by upregulated PD-1/PD-L1 and CD200R/CD200, alongside reduced CTLA-4/CD86 expression. These alterations were consistently reflected at the cell surface, in soluble serum, and at the transcriptomic level, suggesting a global dysregulation of inhibitory pathways that may sustain chronic immune activation in glomerulopathies. Notably, these changes occurred largely independently of patient sex, highlighting their universal character in disease pathophysiology. Although subtle distinctions were identified between IgAN and MPGN, notably in NK and CD8^+^ T cell subpopulations, their overall profiles showed substantial overlap. ROC curve analyses confirmed the diagnostic potential of selected markers, particularly CD19^+^CTLA-4^+^, T-cell PD-1/PD-L1, and soluble PD-1/PD-L1/CD200R/CD200, which effectively distinguished GN from healthy volunteers but were less reliable in differentiating IgAN from MPGN.

Further studies are warranted to validate these findings in larger, multicenter cohorts and to establish standardized thresholds for checkpoint expression and soluble forms as clinical biomarkers. Longitudinal analyses could determine their utility in monitoring disease activity, predicting progression, or assessing treatment response. Mechanistic investigations are also needed to clarify how checkpoint dysregulation contributes to glomerular injury and whether these pathways represent viable therapeutic targets. Given the success of checkpoint modulation in oncology, carefully designed preclinical and clinical studies might explore the potential of immune checkpoint–based interventions in glomerulopathies, while balancing efficacy with the risk of immune-related adverse events. Ultimately, integrating immune checkpoint profiling into nephrology practice may offer a novel, personalized approach to diagnosing, risk-stratifying, and managing patients with GN.

## Figures and Tables

**Figure 2:**
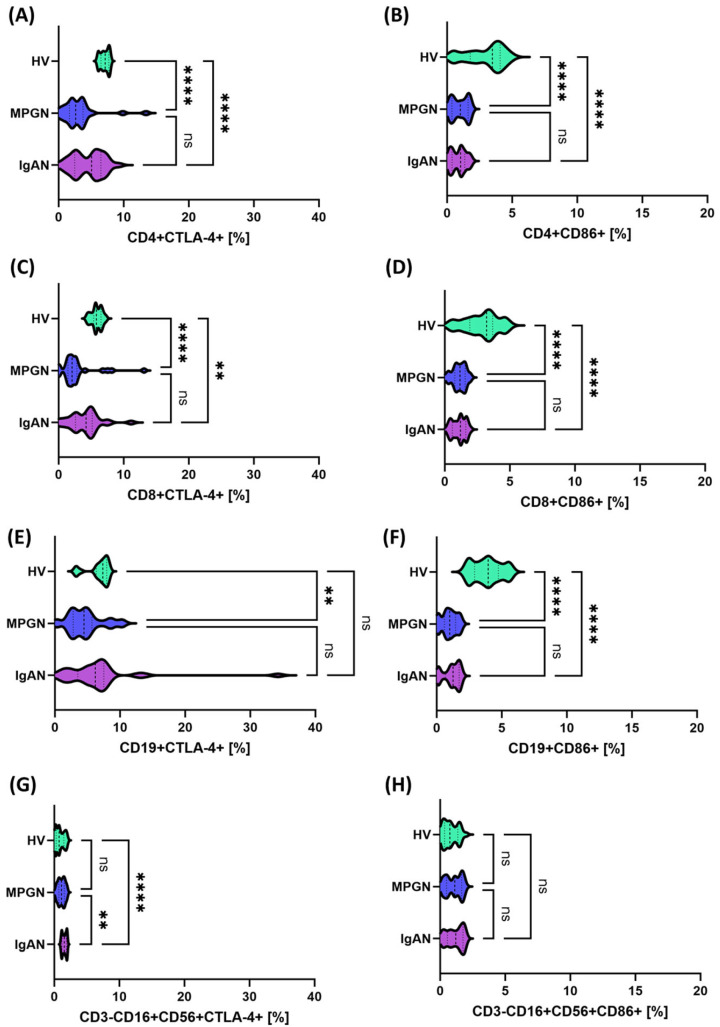
Expression of CTLA-4 and CD86 molecules on the surface of selected immune cell subpopulations in patients with IgAN (purple), MPGN (blue), and healthy volunteers (HV, green). (**A**) CD4+CTLA-4+, (**B**) CD4+CD86+, (**C**) CD8+CTLA-4+, (**D**) CD8+CD86+, (**E**) CD19+CTLA-4+, (**F**) CD19+CD86+, (**G**) CD3-CD16+CD56^+^CTLA-4+, (**H**) CD3-CD16+CD56+CD86+. Data are presented as violin plots with median and quartiles. Statistically significant differences between groups were noted: ** *p* < 0.01, **** *p* < 0.0001, ns—not significant.

**Figure 5:**
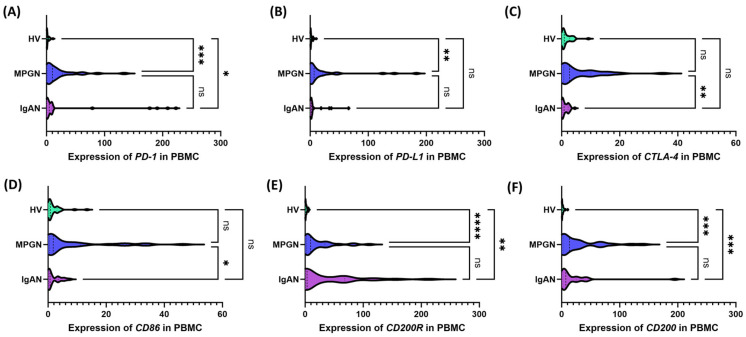
Expression of genes encoding immune checkpoints in peripheral blood mononuclear cells (PBMCs) from patients with IgAN (purple), MPGN (blue), and healthy volunteers (HV, green). (**A**) *PD-1*, (**B**) *PD-L1*, (**C**) *CTLA-4*, (**D**) *CD86*, (**E**) *CD200R*, (**F**) *CD200*. Data are presented as violin plots, showing the median, quartiles, and full distribution. Significant differences between groups are indicated by: * *p* < 0.05, ** *p* < 0.01, *** *p* < 0.001, **** *p* < 0.0001.; ns—not significant.

**Table 1:** Selected laboratory parameters of patients with IgAN, MPGN, and HV.

Parameters		IgAN (*n* = 30)	MPGN (*n* = 30)	HV (*n* = 30)	*p*-Value			
		Mediana (Q1–Q3)	Mediana (Q1–Q3)	Mediana (Q1–Q3)	All	IgAN vs. MPGN	IgAN vs. HV	MPGN vs. HV
Hematological parameters	WBC [10^3^/mm^3^]	7.12 (5.88–9.57)	7.50 (7.00–9.49)	6.30 (5.91–6.80)	0.0003	0.3083	0.0478	0.0002
	NEU [10^3^/mm^3^]	4.79 (4.51–5.04)	6.50 (5.92–7.08)	6.65 (4.82–6.98)	0.0011	0.0013	0.02	>0.9999
	MON [10^3^/mm^3^]	0.59 (0.50–0.60)	0.50 (0.46–0.60)	0.57 (0.43–0.77)	0.2795	0.4564	>0.9999	0.5529
	LYM [10^3^/mm^3^]	2.11 (1.57–2.63)	1.75 (1.47–2.10)	1.95 (1.84–2.21)	0.1634	0.2699	>0.9999	0.331
	EOS [10^3^/mm^3^]	0.19 (0.18–0.20)	0.27 (0.19–0.36)	0.17 (0.12–0.19)	0.0016	0.1614	0.2954	0.001
	BAS [10^3^/mm^3^]	0.10 (0.09–0.10)	0.09 (0.00–0.10)	0.02 (0.00–0.02)	<0.0001	0.0036	<0.0001	0.0058
	RBC [10^6^/mm^3^]	4.23 (3.60–4.55)	4.55 (4.20–5.00)	4.51 (4.24–4.90)	0.0028	0.0055	0.0156	>0.9999
	HGB [g/dL]	12.32 (10.90–13.63)	13.87 (12.90–14.43)	14.76 (13.03–15.70)	0.0002	0.02	0.0002	0.5911
	PLT [10^3^/mm^3^]	213.50 (196.19–273.47)	226.21 (202.77–254.00)	247.00 (212.54–306.00)	0.1381	>0.9999	0.1713	0.4367
Kidney function and protein metabolism	UREA [mg/dL]	44.00 (31.64–51.56)	33.55 (31.93–35.85)	21.00 (18.80–26.75)	<0.0001	0.561	<0.0001	0.0001
	CREATININE [mg/dL]	1.25 (0.90–1.65)	0.68 (0.59–0.85)	0.70 (0.70–0.80)	<0.0001	<0.0001	0.0002	>0.9999
	URIC ACID [mg/dL]	6.27 (4.61–7.47)	7.53 (6.90–7.88)	4.20 (3.60–4.70)	<0.0001	0.0247	<0.0001	<0.0001
	eGFR	47.98 (41.55–56.75)	52.50 (50.00–57.00)	129.00 (126.00–137.00)	<.0001	0.5315	<0.0001	<0.0001
	TOTAL PROTEIN [g/dL]	5.70 (5.33–6.20)	4.62 (4.12–5.15)	7.40 (7.09–7.80)	<0.0001	0.0007	0.0004	<0.0001
	ALBUMIN [g/L]	3.50 (2.98–3.64)	2.93 (2.31–3.39)	4.28 (3.99–4.47)	<0.0001	0.2342	0.0003	<0.0001
	PROTEINURIA [g/24 h]	1.80 (1.27–2.94)	5.00 (4.14–6.40)	0.00 (0.00–0.00)	<0.0001	0.0029	<0.0001	<0.0001
Lipid profile	CHOLESTEROL [mg/dL]	185.50 (170.43–207.74)	209.61 (189.78–289.93)	149.20 (134.25–161.97)	<0.0001	0.0179	0.0003	<0.0001
	TRIGLYCERIDES [mg/dL]	121.44 (98.58–187.75)	159.50 (147.66–175.25)	115.00 (96.75–134.00)	<0.0001	0.1732	0.0433	<0.0001
	HDL [mg/dL]	46.74 (38.54–57.78)	52.84 (50.16–59.13)	60.00 (50.00–70.00)	0.007	0.3464	0.0049	0.3464
	LDL [mg/dL]	103.00 (93.38–122.59)	117.90 (109.78–166.95)	102.00 (93.00–119.00)	0.0006	0.0039	>0.9999	0.0019
Immunoglobulins	IgG [g/L]	7.98 (6.34–9.13)	5.25 (4.08–6.02)	5.20 (4.87–6.15)	<0.0001	<0.0001	<0.0001	>0.9999
	IgM [g/L]	1.10 (0.80–1.37)	1.36 (1.18–1.60)	1.80 (1.13–2.30)	0.0009	0.0499	0.0007	0.577
	IgA [g/L]	3.25 (2.49–4.16)	2.81 (1.76–3.07)	2.42 (1.82–2.94)	0.0081	0.0342	0.0143	>0.9999

Abbreviations: WBC—white blood cells; NEU—neutrophils; MON—monocytes; LYM—lymphocytes; EOS—eosinophils; BAS—basophils; RBC—red blood cells; HGB—hemoglobin; PLT—platelets; UREA—blood urea; CREATININE—serum creatinine; URIC ACID—serum uric acid; eGFR—estimated glomerular filtration rate; CHOLESTEROL—total cholesterol; TRIGLYCERIDES—triglycerides; HDL—high-density lipoprotein cholesterol; LDL—low-density lipoprotein cholesterol; IgG—immunoglobulin G; IgM—immunoglobulin M; IgA—immunoglobulin A; TOTAL PROTEIN—total serum protein; ALBUMIN—serum albumin; PROTEINURIA—24 h urinary protein excretion.

**Table 2:** Analysis of basic lymphocyte subpopulations and CD4+/CD8+ ratio in patients with IgAN, MPGN, and HV.

Parameters	IgAN (*n* = 30)	MPGN (*n* = 30)	HV (*n* = 30)	*p*-Value			
	Mediana (Q1–Q3)	Mediana (Q1–Q3)	Mediana (Q1–Q3)	All	IgAN vs. MPGN	IgAN vs. HV	MPGN vs. HV
CD45+ [%]	98.86 (97.96–99.51)	97.34 (96.12–99.14)	98.65 (91.25–99.68)	0.079	0.073	0.6948	0.8737
CD3+ [%]	74.10 (71.33–78.28)	74.64 (71.60–76.47)	73.16 (59.72–87.98)	0.9489	>0.9999	>0.9999	>0.9999
CD4+ [%]	44.66 (38.85–47.39)	42.31 (35.95–48.31)	42.31 (24.42–63.35)	0.565	>0.9999	>0.9999	>0.9999
CD8+ [%]	24.96 (19.82–29.06)	26.55 (22.36–34.42)	27.11 (17.89–48.68)	0.2697	0.4323	0.5459	>0.9999
CD19+ [%]	11.77 (7.87–14.84)	10.69 (6.42–16.06)	12.36 (1.66–70.30)	0.6345	>0.9999	>0.9999	>0.9999
CD3-CD16+CD56+ [%]	11.61 (8.83–13.23)	11.11 (9.36–16.55)	9.96 (6.63–36.79)	0.4064	0.581	>0.9999	>0.9999
Ratio CD4+/CD8+	1.74 (1.42–2.35)	1.57 (1.08–2.11)	1.58 (0.71–3.24)	0.3492	0.5412	0.7483	>0.9999

**Table 3:** Summary of significant correlations common to IgAN and MPGN (Spearman’s rank coefficient values, R).

Para Zmiennych	R (MPGN)	R (IgAN)
Albumin & IgG	0.509	0.673
Albumin & Total protein	0.603	0.718
Albumin & Urea	0.566	0.384
CD19+CD86+ & CD19+PD-L1+	−0.562	0.370
CD19+CTLA-4+ & CD4+CTLA-4+	0.496	0.601
CD19+CTLA-4+ & CD8+CTLA-4+	0.570	0.374
CD19+PD-1+ & CD19+PD-L1+	0.715	0.475
CD19+PD-L1+ & CD8+PD-1+	0.538	0.362
CD3-CD16+CD56+CTLA-4+ & CD4+CD86+	−0.546	0.430
CD3-CD16+CD56+PD-L1+ & Total protein	0.532	−0.370
CD3-CD16+CD56+PD-L1+ & WBC	−0.365	0.385
CD4+CD200R+ & CD8+PD-1+	0.485	−0.539
CD4+CTLA-4+ & CD8+CTLA-4+	0.466	0.452
CHOLESTEROL & LDL	0.534	0.839
CHOLESTEROL & Proteinuria	0.459	0.379
Creatine & Urea	0.510	0.871
Creatine & Uric acid	0.413	0.609
IgG & Total protein	0.638	0.793
IgM & RBC	−0.427	−0.397
LYM & RBC	−0.495	0.445
PLT & sCD200R	−0.390	0.363
qCD200R & qPD-1	0.439	0.455
qCD86 & qPD-L1	0.383	0.466
sPD-1 & sPD-L1	0.380	0.375

## Data Availability

The original contributions presented in this study are included in the article/Supplementary Material. Further inquiries can be directed to the corresponding author.

## Supplementary Materials

The following supporting information can be downloaded at: https://www.mdpi.com/article/10.3390/cells14191551/s1, Figure S1: Example of a gating strategy for IgA nephropathy and the PD-1 molecule; Figure S2: Expression of PD-1 and PD-L1 on selected immune cell subpopulations in patients with IgA nephropathy (IgAN, purple), MPGN (blue), and healthy volunteers (HV, green) by gender; Figure S3: Expression of CTLA-4 and CD86 molecules on selected immune cell subpopulations, categorized by gender in healthy controls (HV, green), IgAN (purple), and MPGN (blue); Figure S4: Expression of CD200R and CD200 molecules on the surface of selected immune cell subpopulations by gender in healthy volunteers (HV, green), patients with IgAN (purple), and patients with MPGN (blue); Figure S5: Serum concentrations of soluble immune checkpoints by gender in healthy volunteers (HV, green), patients with IgAN (purple), and patients with MPGN (blue); Figure S6: Expression of genes encoding immune checkpoints in peripheral blood mononuclear cells (PBMCs) by gender in patients with IgA nephropathy (IgAN, purple), MPGN (blue), and healthy volunteers (HV, green); Table S1: Expression of immune checkpoints and their ligands on selected lymphocyte subpopulations and NK cells, as well as soluble forms and transcript levels (qPCR) in patients with IgA nephropathy (IgAN), MPGN and healthy volunteers (HV); Table S2: Hematological and biochemical parameters across the study groups; Table S3: Comparison of the studied parameters between genders and in the patient groups compared to the control group; Table S4: Statistically significant correlations between clinical and immunological parameters in patients with IgAN (Spearman rank analysis); Table S5: Statistically significant correlations between clinical and immunological parameters in patients with MPGN (Spearman rank analysis); Table S6: Receiver Operating Characteristic (ROC) curve analysis for determining the sensitivity and specificity of the parameters studied and their ability to distinguish individual disease entities from each other and from healthy volunteers.

## Abbreviations

**Abbreviation**	**Full Term**
IgAN	IgA nephropathy
MPGN	Membranoproliferative glomerulonephritis
HV	Healthy volunteers
PD-1	Programmed cell death protein 1
PD-L1	Programmed death-ligand 1
CTLA-4	Cytotoxic T-lymphocyte–associated protein 4
CD86	Cluster of differentiation 86
CD200R	CD200 receptor
CD200	Cluster of differentiation 200
PBMC	Peripheral blood mononuclear cells
ROC	Receiver operating characteristic
sPD-1	Soluble PD-1
sPD-L1	Soluble PD-L1
sCTLA-4	Soluble CTLA-4
sCD86	Soluble CD86
sCD200	Soluble CD200
sCD200R	Soluble CD200R
GN	Glomerulonephritis
NK	Natural killer (cells)
ELISA	Enzyme-linked immunosorbent assay
CD4^+^/CD8^+^ T cells	T lymphocyte subsets expressing CD4 or CD8
CD19^+^ B cells	B lymphocytes expressing CD19
