# Supplementary material for: Checkpoint Imbalance in Primary Glomerulopathies: Comparative Insights into IgA Nephropathy and Membranoproliferative Glomerulonephritis
Source: Cells. 2025 Oct 3;14(19):1551. doi: 10.3390/cells14191551 (PMC12524032; doi:10.3390/cells14191551)
Supplement: Supplementary file 1 [file cells-14-01551-s001.zip › cells-3893184-supplementary.pdf]

Supplementary Materials Table S1- Expression of immune checkpoints and their ligands on selected lymphocyte subpopulations and NK cells, as well as soluble forms and transcript levels (qPCR) in patients with IgA nephropathy (IgAN), MPGN and healthy volunteers (HV).

	IgAN (n=30)	MPGN (n=30)	HV (n=30)	p-Value			
	Mediana (Q1-Q3)	Mediana (Q1-Q3)	Mediana (Q1-Q3)	All	IgAN vs. MPGN	IgAN vs. HV	MPGN vs. HV
CD4+PD-1+ [%]	15.00 (11.84-17.84)	12.54 (9.99-14.39)	0.87 (0.31-3.56)	<0.0001	0.8981	<0.0001	<0.0001
CD8+PD-1+ [%]	12.54 (10.52-18.16)	11.31 (6.81-13.59)	1.56 (0.41-5.75)	<0.0001	0.5888	<0.0001	<0.0001
CD19+PD-1+ [%]	2.49 (1.54-5.67)	5.27 (0.95-6.93)	0.25 (0.04-1.74)	<0.0001	>0.9999	<0.0001	<0.0001
CD3-CD16+CD56+ PD-1 [%]	4.17 (3.32-5.16)	5.08 (4.27-6.14)	2.89 (2.48-3.26)	<0.0001	0.0208	0.0014	<0.0001
CD4+PD-L1+ [%]	15.49 (10.77-18.75)	8.59 (5.70-13.56)	1.12 (0.25-1.99)	<0.0001	0.1637	<0.0001	<0.0001
CD8+PD-L1+ [%]	7.21 (5.45-8.80)	14.62 (9.82-18.09)	0.47 (0.11-1.46)	<0.0001	0.0034	<0.0001	<0.0001
CD19+PD-L1+ [%]	11.08 (3.64-12.72)	5.46 (2.41-7.28)	0.59 (0.11-1.89)	<0.0001	0.0755	<0.0001	<0.0001
CD3-CD16+CD56+PD-L1 [%]	3.38 (2.61-4.22)	3.82 (3.15-4.80)	3.53 (2.60-4.19)	0.0771	0.1493	>0.9999	0.1502
CD4+CTLA-4+ [%]	5.06 (2.49-6.34)	2.61 (1.78-3.69)	7.16 (6.03-7.95)	<0.0001	0.1045	<0.0001	<0.0001
CD8+CTLA-4+ [%]	4.22 (2.72-5.13)	2.05 (1.44-2.77)	5.78 (4.34-7.31)	<0.0001	0.0983	0.0015	<0.0001
CD19+CTLA-4+ [%]	6.19 (3.89-7.36)	4.45 (2.88-5.56)	7.34 (3.13-8.27)	0.0021	0.2773	0.2013	0.0013
CD3-CD16+CD56+CTLA-4+ [%]	1.52 (1.18-1.83)	1.09 (0.71-1.50)	0.73 (0.30-1.44)	<0.0001	0.0082	<0.0001	0.6121
CD4+CD86+ [%]	1.03 (0.39-1.32)	1.01 (0.39-1.58)	3.47 (0.40-5.00)	<0.0001	>0.9999	<0.0001	<0.0001
CD8+CD86+ [%]	1.20 (0.63-1.57)	1.16 (0.79-1.53)	3.21 (0.41-4.90)	<0.0001	>0.9999	<0.0001	<0.0001

CD19+CD86+ [%]	1.26 (0.78-1.70)	0.99 (0.61-1.43)	3.96 (2.21-5.68)	<0.0001	>0.9999	<0.0001	<0.0001
CD3-CD16+CD56+CD86+ [%]	1.20 (0.61-1.73)	1.12 (0.52-1.65)	0.74 (0.34-1.32)	0.0927	>0.9999	0.1064	0.3621
CD4+CD200+ [%]	49.82 (40.93-55.46)	39.15 (33.28-56.05)	4.76 (4.06-5.80)	<0.0001	0.7186	<0.0001	<0.0001
CD8+CD200+ [%]	38.33 (31.29-44.95)	27.10 (20.10-35.65)	5.15 (4.17-5.97)	<0.0001	0.0622	<0.0001	<0.0001
CD19+CD200+ [%]	35.89 (28.90-41.61)	34.12 (29.70-38.21)	5.08 (4.01-5.94)	<0.0001	>0.9999	<0.0001	<0.0001
CD3-CD16+CD56+CD200+ [%]	8.05 (7.39-8.86)	8.13 (7.53-9.07)	5.67 (4.80-6.30)	<0.0001	>0.9999	<0.0001	<0.0001
CD4+CD200R+ [%]	59.07 (51.87-71.71)	60.20 (52.66-66.76)	7.30 (3.37-9.62)	<0.0001	>0.9999	<0.0001	<0.0001
CD8+CD200R+ [%]	53.11 (43.22-58.08)	49.05 (43.62-53.12)	7.68 (4.26-9.75)	<0.0001	>0.9999	<0.0001	<0.0001
CD19+CD200R+ [%]	50.98 (43.14-65.14)	53.46 (42.85-60.08)	27.46 (10.15-39.56)	<0.0001	>0.9999	<0.0001	<0.0001
CD3-CD16+CD56+CD200R+ [%]	8.70 (7.96-9.39)	7.01 (6.48-8.32)	6.40 (5.44-7.30)	0.0001	0.0001	<0.0001	0.0388
sPD-1	27.61 (24.10-30.50)	24.67 (20.56-27.29)	2.60 (2.41-2.68)	<0.0001	0.1831	<0.0001	<0.0001
sPD-L1	31.56 (24.87-32.81)	27.94 (21.14-29.61)	1.79 (1.66-1.85)	<0.0001	0.1282	<0.0001	<0.0001
sCTLA-4	7.58 (6.24-8.77)	6.73 (5.47-7.83)	4.68 (4.41-5.07)	<0.0001	0.2184	<0.0001	<0.0001
sCD86	3.99 (3.55-4.32)	4.46 (3.96-5.21)	6.00 (5.31-6.47)	<0.0001	0.082	<0.0001	<0.0001
sCD200	33.28 (31.29-36.76)	30.03 (27.30-32.61)	4.07 (3.84-4.41)	<0.0001	0.0314	<0.0001	<0.0001
sCD200R	34.48 (31.29-36.44)	30.81 (27.93-32.61)	3.60 (3.40-3.90)	<0.0001	0.0552	<0.0001	<0.0001

qPD-1	4.57 (0.68-9.39)	10.05 (1.50-24.99)	0.74 (0.40-3.57)	<0.0001	0.5585	0.043	0.0005
qPD-L1	2.03 (0.80-3.25)	6.57 (0.96-20.28)	0.81 (0.52-1.79)	0.0048	0.2127	0.4347	0.0033
qCTLA-4	0.82 (0.22-2.16)	2.68 (0.77-9.83)	0.92 (0.43-2.50)	0.0065	0.0061	>0.9999	0.0868
qCD86	0.66 (0.16-3.35)	1.74 (0.56-10.08)	0.80 (0.40-2.97)	0.0262	0.0244	>0.9999	0.2257
qCD200	7.06 (2.11-23.92)	13.26 (2.64-36.14)	1.00 (0.48-2.20)	<0.0001	>0.9999	<0.0001	<0.0001
qCD200R	3.32 (0.72-65.77)	9.28 (2.47-32.55)	1.11 (0.50-2.39)	<0.0001	0.7098	0.0065	<0.0001

Supplementary Materials Table S2 - Hematological and biochemical parameters across the study groups

	IgAN Male (n=17)	IgAN Female (n=13)	MPGN Male (n=17)	MPGN Female (n=13)	HV Male (n=17)	HV Female (n=13)	p-Value														
	Mediana (Q1-Q3)	Mediana (Q1-Q3)	Mediana (Q1-Q3)	Mediana (Q1-Q3)	Mediana (Q1-Q3)	Mediana (Q1-Q3)	IgAN Male vs. IgAN Female	IgAN Male vs. MPGN Male	IgAN Male vs. MPGN Female	IgAN Male vs. HV Male	IgAN Male vs. HV Female	IgAN Female vs. MPGN Male	IgAN Female vs. MPGN Female	IgAN Female vs. HV Male	IgAN Female vs. HV Female	MPGN Male vs. MPGN Female	MPGN Male vs. HV Male	MPGN Male vs. HV Female	MPGN Female vs. HV Male	MPGN Female vs. HV Female	HV Male vs. HV Female
Age	48.00 (41.00-51.00)	39.00 (32.00-46.00)	48.50 (40.50-53.75)	48.00 (38.00-54.00)	48.00 (44.00-55.00)	41.00 (39.00-44.00)	0.9836	>0.9999	>0.9999	>0.9999	>0.9999	>0.9999	>0.9999	0.1212	>0.9999	>0.9999	>0.9999	>0.9999	>0.9999	>0.9999	0.4515

WBC [10^3/mm^3]	7.00 (5.20-8.70)	7.23 (6.84-9.57)	7.24 (6.98-8.20)	9.21 (7.55-10.28)	6.30 (5.90-6.80)	6.30 (6.00-6.80)	>0.9999	>0.9999	0.1904	>0.9999	>0.9999	>0.9999	>0.9999	0.1454	0.1652	>0.9999	0.1229	0.1516	0.0128	0.0165	>0.9999
NEU [10^3/mm^3]	4.90 (4.30-5.05)	4.77 (4.51-5.00)	6.50 (5.53-6.88)	6.65 (6.12-7.26)	5.90 (4.70-6.70)	6.90 (6.80-7.25)	>0.9999	0.1289	0.055	>0.9999	0.0333	0.5629	0.2122	>0.9999	0.1602	>0.9999	>0.9999	>0.9999	>0.9999	>0.9999	>0.9999
MON [10^3/mm^3]	0.60 (0.55-0.70)	0.55 (0.46-0.57)	0.50 (0.46-0.60)	0.52 (0.47-0.60)	0.65 (0.50-0.79)	0.50 (0.40-0.56)	0.5716	0.3115	>0.9999	>0.9999	0.228	>0.9999	>0.9999	0.4013	>0.9999	>0.9999	0.2026	>0.9999	>0.9999	>0.9999	0.153
LYM [10^3/mm^3]	2.30 (1.90-2.67)	1.75 (1.32-2.48)	1.67 (1.48-2.03)	1.95 (1.52-2.23)	1.92 (1.50-2.10)	1.97 (1.90-2.50)	>0.9999	0.2372	>0.9999	>0.9999	>0.9999	>0.9999	>0.9999	>0.9999	>0.9999	>0.9999	>0.9999	0.4955	>0.9999	>0.9999	>0.9999
EOS [10^3/mm^3]	0.20 (0.18-0.20)	0.18 (0.17-0.20)	0.30 (0.20-0.37)	0.19 (0.00-0.27)	0.17 (0.10-0.20)	0.16 (0.13-0.18)	>0.9999	0.5422	>0.9999	>0.9999	>0.9999	0.1006	>0.9999	>0.9999	>0.9999	0.2336	0.007	0.0024	>0.9999	>0.9999	>0.9999
BAS [10^3/mm^3]	0.10 (0.10-0.10)	0.09 (0.08-0.10)	0.09 (0.00-0.10)	0.09 (0.01-0.10)	0.02 (0.01-0.02)	0.02 (0.00-0.02)	>0.9999	0.0092	0.1768	<0.0001	<0.0001	>0.9999	>0.9999	0.0053	0.0139	>0.9999	0.3048	0.5372	0.3776	0.564	>0.9999

RBC [10^6/mm^3]	4.23 (4.00-4.60)	4.23 (3.28-4.40)	4.50 (4.20-4.93)	4.62 (4.19-5.00)	4.40 (4.10-4.93)	4.51 (4.45-4.79)	>0.9999	0.7627	>0.9999	>0.9999	0.5716	0.1125	0.2095	0.5822	0.0938	>0.9999	>0.9999	>0.9999	>0.9999	>0.9999	>0.9999
HGB [g/dl]	12.90 (11.87-13.80)	11.96 (9.75-13.00)	13.92 (13.27-14.58)	13.68 (12.49-14.15)	13.10 (12.78-15.70)	14.90 (14.00-15.00)	>0.9999	>0.9999	>0.9999	>0.9999	0.0439	0.0232	0.8283	0.0358	0.0003	>0.9999	>0.9999	>0.9999	>0.9999	0.6324	>0.9999
PLT [10^3/mm^3]	214.00 (204.00-265.88)	212.00 (195.90-276.00)	229.71 (202.02-252.00)	222.11 (211.66-265.49)	231.00 (209.62-282.00)	254.00 (245.00-308.00)	>0.9999	>0.9999	>0.9999	>0.9999	0.7776	>0.9999	>0.9999	>0.9999	0.7444	>0.9999	>0.9999	>0.9999	>0.9999	>0.9999	>0.9999
UREA [mg/dl]	40.99 (31.78-62.99)	44.30 (31.60-49.20)	35.40 (32.07-36.07)	32.26 (27.52-33.20)	20.00 (18.80-24.00)	25.00 (18.80-27.00)	>0.9999	>0.9999	>0.9999	0.0001	0.0024	>0.9999	>0.9999	0.0022	0.0175	>0.9999	0.0034	0.0327	0.2755	0.7852	>0.9999
CREATININE [mg/dl]	1.24 (0.89-1.87)	1.32 (0.92-1.65)	0.68 (0.61-0.79)	0.73 (0.57-1.00)	0.70 (0.70-0.80)	0.75 (0.70-0.80)	>0.9999	0.0018	0.1266	0.0579	0.1461	0.0014	0.0773	0.0362	0.0889	>0.9999	>0.9999	>0.9999	>0.9999	>0.9999	>0.9999
URIC ACID [mg/dl]	6.50 (4.90-7.60)	6.20 (4.51-6.90)	7.52 (6.77-7.78)	7.55 (7.38-7.99)	4.20 (3.60-4.80)	3.95 (3.60-4.30)	>0.9999	>0.9999	0.719	0.0194	0.0089	0.7189	0.2543	0.24	0.1115	>0.9999	<0.0001	<0.0001	<0.0001	<0.0001	>0.9999

eGFR	48.12 (43.70-57.00)	43.00 (41.40-52.62)	52.50 (50.04-59.47)	52.18 (46.87-56.60)	134.40 (126.00-137.00)	129.00 (126.00-137.00)	>0.9999	>0.9999	>0.9999	<0.0001	<0.0001	>0.9999	>0.9999	<0.0001	<0.0001	>0.9999	<0.0001	0.0006	0.0004	0.0015	>0.9999
CHOLESTEROL [mg/dl]	186.90 (173.00-209.00)	174.00 (170.20-195.00)	209.61 (191.08-239.25)	214.29 (192.50-333.61)	157.00 (139.00-169.00)	147.00 (133.00-157.00)	>0.9999	>0.9999	>0.9999	0.0657	0.0068	0.324	0.4116	0.7512	0.1234	>0.9999	<0.0001	<0.0001	0.0005	<0.0001	>0.9999
TRIGLYCERIDES [mg/dl]	134.00 (97.92-197.00)	109.35 (100.56-160.00)	161.49 (151.55-169.75)	152.03 (143.04-202.96)	79.00 (69.00-120.00)	133.00 (120.00-146.00)	>0.9999	>0.9999	>0.9999	0.0441	>0.9999	>0.9999	>0.9999	0.4596	>0.9999	>0.9999	0.0002	0.5029	0.0081	>0.9999	>0.9999
HDL [mg/dl]	43.71 (35.29-57.10)	52.00 (43.00-59.17)	51.72 (49.98-58.04)	53.56 (51.70-64.07)	64.00 (51.70-73.00)	55.00 (50.00-64.00)	>0.9999	>0.9999	0.9858	0.0156	0.6141	>0.9999	>0.9999	0.5259	>0.9999	>0.9999	0.7037	>0.9999	>0.9999	>0.9999	>0.9999
LDL [mg/dl]	105.20 (94.76-116.06)	101.36 (72.00-124.77)	117.90 (109.93-153.33)	134.97 (104.40-166.95)	102.00 (90.00-120.00)	104.00 (97.00-112.80)	>0.9999	0.2509	0.6713	>0.9999	>0.9999	0.2103	0.5283	>0.9999	>0.9999	>0.9999	0.0855	0.2822	0.3092	0.6589	>0.9999

IgG [g/l]	7.31 (6.32-9.12)	8.28 (7.86-9.13)	5.35 (3.69-5.90)	5.11 (4.36-6.55)	5.52 (4.89-6.49)	5.12 (4.83-5.50)	>0.9999	0.0004	0.032	0.0292	0.0016	0.0015	0.0589	0.0646	0.0046	>0.9999	>0.9999	>0.9999	>0.9999	>0.9999	>0.9999
IgM [g/l]	1.20 (0.80-1.40)	1.07 (0.83-1.16)	1.40 (1.19-1.65)	1.30 (1.13-1.48)	1.80 (1.10-2.30)	1.50 (1.20-2.16)	>0.9999	>0.9999	>0.9999	0.1442	0.6587	0.2367	>0.9999	0.0189	0.1183	>0.9999	>0.9999	>0.9999	>0.9999	>0.9999	>0.9999
IgA [g/l]	3.08 (2.49-4.18)	3.83 (2.58-4.09)	2.89 (2.20-3.18)	1.97 (0.94-2.65)	2.44 (1.87-3.00)	2.30 (1.80-2.60)	>0.9999	>0.9999	0.0893	>0.9999	0.2815	>0.9999	0.0146	0.9045	0.0476	0.4128	>0.9999	>0.9999	>0.9999	>0.9999	>0.9999
TOTAL PROTEIN [g/dl]	5.70 (5.40-6.00)	5.70 (5.24-6.20)	4.84 (4.37-5.36)	4.28 (4.03-4.84)	7.35 (6.99-7.60)	7.80 (7.24-7.88)	>0.9999	0.1324	0.0413	0.1467	0.0535	0.3497	0.1028	0.1586	0.0598	>0.9999	<0.0001	<0.0001	<0.0001	<0.0001	>0.9999
ALBUMIN [g/l]	3.30 (3.04-3.60)	3.59 (2.96-3.65)	2.96 (2.37-3.43)	2.84 (2.20-3.16)	4.26 (3.70-4.49)	4.28 (4.18-4.40)	>0.9999	>0.9999	>0.9999	0.1385	0.0234	>0.9999	>0.9999	0.2198	0.0421	>0.9999	0.0017	0.0002	0.0023	0.0003	>0.9999
PROTEINURIA [g/24h]	1.86 (1.24-2.95)	1.66 (1.36-2.16)	4.85 (4.09-5.88)	5.35 (4.53-6.40)	0.00 (0.00-0.00)	0.00 (0.00-0.00)	>0.9999	0.1809	0.4193	0.0014	0.0042	0.2583	0.4961	0.0052	0.0118	>0.9999	<0.0001	<0.0001	<0.0001	<0.0001	>0.9999

Supplementary Materials Table S3 – Comparison of the studied parameters between genders and in the patient groups compared to the control group.

	IgAN Male (n=17)	IgAN Female (n=13)	MPGN Male (n=17)	MPGN Female (n=13)	HV Male (n=17)	HV Female (n=13)	p-Value														
	Mediana (Q1-Q3)	Mediana (Q1-Q3)	Mediana (Q1-Q3)	Mediana (Q1-Q3)	Mediana (Q1-Q3)	Mediana (Q1-Q3)	IgAN Male vs. IgAN Female	IgAN Male vs. MPGN Male	IgAN Male vs. MPGN Female	IgAN Male vs. HV Male	IgAN Male vs. HV Female	IgAN Female vs. MPGN Male	IgAN Female vs. MPGN Female	IgAN Female vs. HV Male	IgAN Female vs. HV Female	MPGN Male vs. MPGN Female	MPGN Male vs. HV Male	MPGN Male vs. HV Female	MPGN Female vs. HV Male	MPGN Female vs. HV Female	HV Male vs. HV Female
CD45+ [%]	99.46 (98.47-99.56)	98.00 (96.00-98.86)	97.75 (96.65-99.24)	97.33 (95.11-97.87)	99.03 (97.07-99.46)	97.36 (97.18-98.82)	0.0649	0.0824	0.0200	>0.9999	0.0863	>0.9999	>0.9999	>0.9999	>0.9999	>0.9999	>0.9999	>0.9999	>0.9999	>0.9999	>0.9999
CD3+ [%]	73.34 (72.06-83.73)	74.58 (70.34-77.75)	74.70 (71.72-76.50)	73.78 (71.21-76.17)	75.21 (72.98-79.03)	70.86 (67.52-71.75)	>0.9999	>0.9999	>0.9999	>0.9999	0.7037	>0.9999	>0.9999	>0.9999	>0.9999	>0.9999	>0.9999	0.8443	>0.9999	>0.9999	0.1430
CD4+ [%]	46.31 (42.84-49.78)	39.55 (38.75-46.26)	42.31 (35.95-47.92)	40.75 (34.89-49.28)	47.08 (37.09-49.78)	39.08 (26.13-44.87)	>0.9999	>0.9999	>0.9999	>0.9999	0.9926	>0.9999	>0.9999	>0.9999	>0.9999	>0.9999	>0.9999	>0.9999	>0.9999	>0.9999	>0.9999

CD4+PD-1+ [%]	Ratio CD4+/CD8+	CD3-CD16+CD56+ [%]	CD19+ [%]	CD8+ [%]
13.38 (11.78-16.88)	1.80 (1.47-2.37)	9.78 (9.16-12.92)	12.92 (7.64-14.84)	24.51 (19.56-26.32)
15.73 (12.32-18.72)	1.62 (1.34-2.00)	11.89 (8.22-13.98)	10.86 (8.30-15.02)	25.47 (19.82-29.06)
11.82 (10.45-13.68)	1.58 (1.06-1.92)	14.49 (10.22-17.50)	10.12 (5.43-15.15)	26.88 (25.43-34.90)
13.51 (10.44-15.82)	1.50 (1.14-2.72)	9.79 (8.93-11.34)	11.76 (7.79-16.06)	23.93 (20.19-32.61)
0.99 (0.66-2.27)	1.80 (1.08-2.27)	10.14 (7.59-13.74)	11.90 (6.97-14.84)	26.32 (20.76-34.42)
0.77 (0.53-1.65)	1.44 (0.87-1.80)	9.34 (9.19-23.54)	12.81 (8.94-13.95)	27.11 (24.74-29.98)
>0.9999	>0.9999	>0.9999	>0.9999	>0.9999
>0.9999	>0.9999	0.7359	>0.9999	0.8936
>0.9999	>0.9999	>0.9999	>0.9999	>0.9999
<0.0001	>0.9999	>0.9999	>0.9999	>0.9999
<0.0001	>0.9999	>0.9999	>0.9999	>0.9999
>0.9999	>0.9999	>0.9999	>0.9999	>0.9999
>0.9999	>0.9999	>0.9999	>0.9999	>0.9999
<0.0001	>0.9999	>0.9999	>0.9999	>0.9999
<0.0001	>0.9999	>0.9999	>0.9999	>0.9999
>0.9999	>0.9999	>0.9999	>0.9999	>0.9999
0.0002	>0.9999	>0.9999	>0.9999	>0.9999
0.0001	>0.9999	>0.9999	>0.9999	>0.9999
0.0003	>0.9999	>0.9999	>0.9999	>0.9999
0.0002	>0.9999	>0.9999	>0.9999	>0.9999
>0.9999	>0.9999	>0.9999	>0.9999	>0.9999

CD8+PD-1+ [%]	12.47 (6.76-16.15)	12.56 (11.93-18.61)	11.63 (6.22-13.75)	10.38 (7.93-12.92)	1.65 (1.03-2.60)	1.47 (0.88-2.03)	>0.9999	>0.9999	>0.9999	<0.0001	<0.0001	>0.9999	>0.9999	<0.0001	<0.0001	>0.9999	0.0003	0.0001	0.0168	0.0071	>0.9999
CD19+PD-1+ [%]	1.99 (1.06-2.76)	3.80 (2.21-7.73)	2.57 (0.96-6.31)	5.94 (1.84-7.32)	0.27 (0.12-0.68)	0.24 (0.11-0.41)	>0.9999	>0.9999	>0.9999	0.0025	0.0021	>0.9999	>0.9999	<0.0001	<0.0001	>0.9999	0.0005	0.0005	0.0013	0.0010	>0.9999
CD3-CD16+CD56+ PD-1 [%]	4.39 (3.84-5.61)	3.49 (2.31-4.15)	5.01 (3.74-5.69)	5.42 (4.44-6.30)	2.84 (2.46-3.30)	3.02 (2.58-3.17)	0.5899	>0.9999	>0.9999	0.0037	0.0158	0.0499	0.0322	>0.9999	>0.9999	>0.9999	<0.0001	0.0004	0.0001	0.0005	>0.9999
CD4+PD-L1+ [%]	14.47 (10.47-17.53)	17.43 (15.97-19.23)	6.96 (5.34-12.21)	13.12 (6.67-14.42)	1.05 (0.85-1.49)	1.25 (0.69-1.53)	>0.9999	>0.9999	>0.9999	<0.0001	<0.0001	0.2289	>0.9999	<0.0001	<0.0001	>0.9999	0.0007	0.0009	0.0007	0.0008	>0.9999
CD8+PD-L1+ [%]	7.11 (5.29-7.82)	7.86 (6.33-9.16)	14.30 (9.97-18.07)	14.81 (7.91-17.72)	0.54 (0.34-0.73)	0.37 (0.28-0.49)	>0.9999	0.1040	0.8546	0.0220	0.0059	0.2093	>0.9999	0.0403	0.0114	>0.9999	<0.0001	<0.0001	<0.0001	<0.0001	>0.9999

CD19+PD-L1+ [%]	10.87 (2.84-12.92)	11.29 (9.19-12.70)	5.43 (2.03-7.55)	5.66 (3.72-7.15)	0.78 (0.42-1.07)	0.49 (0.33-0.71)	>0.9999	>0.9999	>0.9999	<0.0001	<0.0001	0.5098	>0.9999	<0.0001	<0.0001	>0.9999	0.0072	0.0017	0.0192	0.0052	>0.9999
CD3-CD16+CD56+PD-L1 [%]	3.02 (2.51-4.33)	3.45 (2.97-4.05)	3.82 (3.30-4.85)	3.55 (2.81-4.56)	3.77 (2.75-4.20)	3.19 (2.59-4.13)	>0.9999	0.3453	>0.9999	>0.9999	>0.9999	>0.9999	>0.9999	>0.9999	>0.9999	>0.9999	>0.9999	0.7592	>0.9999	>0.9999	>0.9999
CD4+CTLA-4+ [%]	4.83 (2.40-6.00)	5.28 (2.92-6.93)	3.04 (1.38-3.87)	2.27 (2.04-3.20)	6.97 (6.61-7.56)	7.33 (6.53-7.83)	>0.9999	>0.9999	>0.9999	0.0122	0.0066	0.9807	0.5264	0.3010	0.1579	>0.9999	<0.0001	<0.0001	0.0002	0.0001	>0.9999
CD8+CTLA-4+ [%]	3.01 (1.94-5.20)	4.23 (3.75-5.00)	1.90 (1.35-3.13)	2.46 (2.04-2.73)	6.35 (5.63-6.58)	5.65 (5.45-5.86)	>0.9999	>0.9999	>0.9999	0.0226	0.3482	>0.9999	0.8679	0.2112	>0.9999	>0.9999	<0.0001	0.0078	0.0003	0.0085	>0.9999
CD19+CTLA-4+ [%]	6.19 (3.70-8.00)	6.19 (5.03-7.26)	4.64 (2.90-7.18)	4.14 (2.63-4.65)	7.25 (6.36-7.86)	7.57 (6.62-8.14)	>0.9999	>0.9999	0.4224	>0.9999	>0.9999	>0.9999	>0.9999	>0.9999	>0.9999	>0.9999	0.4181	0.6880	0.0183	0.0365	>0.9999
CD3-CD16+CD56+CTLA-4+ [%]	1.70 (1.33-1.92)	1.42 (1.14-1.69)	1.32 (0.91-1.64)	0.77 (0.60-0.98)	0.70 (0.30-1.12)	0.74 (0.30-1.62)	>0.9999	0.6457	0.0048	0.0004	0.0985	>0.9999	0.1431	0.0434	>0.9999	0.7126	0.2825	>0.9999	>0.9999	>0.9999	>0.9999

CD4+CD86+ [%]	1.03 (0.32-1.36)	1.02 (0.39-1.17)	0.52 (0.30-1.34)	1.43 (1.15-1.66)	2.26 (1.07-3.56)	3.84 (3.68-4.25)	>0.9999	>0.9999	>0.9999	0.0534	<0.0001	>0.9999	>0.9999	0.0849	<0.0001	0.7044	0.0086	<0.0001	>0.9999	0.0691	0.3762
CD8+CD86+ [%]	1.22 (0.83-1.46)	1.09 (0.59-1.60)	1.10 (0.80-1.48)	1.20 (0.79-1.52)	3.23 (2.30-4.51)	2.98 (2.02-3.31)	>0.9999	>0.9999	>0.9999	0.0035	0.0143	>0.9999	>0.9999	0.0042	0.0147	>0.9999	0.0007	0.0042	0.0265	0.0649	>0.9999
CD19+CD86+ [%]	1.12 (0.49-1.54)	1.45 (1.04-1.74)	1.04 (0.69-1.42)	0.78 (0.58-1.42)	3.44 (2.46-3.97)	4.71 (3.74-5.38)	>0.9999	>0.9999	>0.9999	<0.0001	<0.0001	>0.9999	>0.9999	0.0042	0.0004	>0.9999	<0.0001	<0.0001	0.0002	<0.0001	>0.9999
CD3-CD16+CD56+CD86+ [%]	0.97 (0.47-1.53)	1.50 (0.81-1.85)	1.18 (0.62-1.66)	0.80 (0.40-1.39)	0.81 (0.59-1.28)	0.36 (0.21-1.46)	>0.9999	>0.9999	>0.9999	>0.9999	>0.9999	>0.9999	0.9333	0.9389	0.0964	>0.9999	>0.9999	0.4158	>0.9999	>0.9999	>0.9999
CD4+CD200+ [%]	47.12 (40.21-57.75)	50.92 (44.29-55.35)	39.45 (32.08-76.54)	39.15 (37.50-42.25)	4.76 (4.67-5.12)	4.64 (4.27-4.99)	>0.9999	>0.9999	>0.9999	<0.0001	<0.0001	>0.9999	>0.9999	<0.0001	<0.0001	>0.9999	<0.0001	<0.0001	0.0143	0.0082	>0.9999

CD8+CD200+ [%]	39.67 (32.86-45.14)	37.24 (30.63-44.37)	29.80 (24.74-37.40)	20.32 (18.94-24.70)	5.08 (4.46-5.41)	5.19 (4.53-5.51)	>0.9999	>0.9999	0.1986	<0.0001	<0.0001	>0.9999	0.4040	<0.0001	<0.0001	>0.9999	<0.0001	0.0001	0.0994	0.2186	>0.9999
CD19+CD200+ [%]	36.65 (30.22-46.67)	31.90 (27.20-40.47)	32.71 (29.54-38.57)	35.29 (32.82-37.24)	5.12 (4.81-5.48)	4.87 (4.54-5.23)	>0.9999	>0.9999	>0.9999	<0.0001	<0.0001	>0.9999	>0.9999	0.0002	0.0002	>0.9999	<0.0001	<0.0001	0.0003	0.0002	>0.9999
CD3-CD16+CD56+CD200+ [%]	7.46 (6.63-8.22)	8.62 (7.81-9.24)	8.06 (7.18-9.02)	8.30 (7.85-9.06)	5.57 (4.87-6.35)	5.88 (4.78-6.23)	>0.9999	>0.9999	>0.9999	0.0022	0.0097	>0.9999	>0.9999	<0.0001	<0.0001	>0.9999	<0.0001	0.0004	0.0001	0.0007	>0.9999
CD4+CD200R+ [%]	61.22 (55.52-70.00)	52.57 (46.93-72.28)	60.35 (52.96-67.36)	58.86 (53.33-64.32)	7.80 (5.39-8.66)	7.18 (6.51-8.08)	>0.9999	>0.9999	>0.9999	<0.0001	<0.0001	>0.9999	>0.9999	0.0006	0.0008	>0.9999	<0.0001	<0.0001	0.0005	0.0006	>0.9999
CD8+CD200R+ [%]	55.86 (42.97-60.39)	49.93 (43.96-53.91)	49.05 (46.07-52.41)	48.78 (43.02-53.95)	7.68 (5.76-8.91)	7.68 (6.11-9.45)	>0.9999	>0.9999	>0.9999	<0.0001	<0.0001	>0.9999	>0.9999	<0.0001	0.0004	>0.9999	<0.0001	<0.0001	0.0006	0.0027	>0.9999

CD19+CD200R+ [%]	54.46 (43.57-64.91)	47.68 (39.93-70.98)	55.04 (42.94-62.28)	51.66 (38.72-54.14)	30.73 (23.76-35.19)	22.04 (11.22-34.43)	>0.9999	>0.9999	>0.9999	<0.0001	<0.0001	>0.9999	>0.9999	0.0017	0.0007	>0.9999	0.0002	<0.0001	0.0430	0.0173	>0.9999
CD3-CD16+CD56+CD200R+ [%]	8.92 (7.96-9.40)	8.67 (7.97-9.23)	6.99 (6.47-8.33)	7.11 (6.57-8.11)	6.39 (5.44-7.09)	6.41 (5.44-7.61)	>0.9999	0.0256	0.0811	<0.0001	0.0002	0.0578	0.1307	<0.0001	0.0006	>0.9999	0.4019	>0.9999	>0.9999	>0.9999	>0.9999
sPD-1	25.95 (24.15-30.33)	29.27 (21.98-31.25)	25.63 (20.44-27.46)	23.33 (20.97-27.13)	2.52 (2.40-2.68)	2.60 (2.44-2.68)	>0.9999	>0.9999	>0.9999	<0.0001	<0.0001	>0.9999	>0.9999	<0.0001	<0.0001	>0.9999	<0.0001	0.0008	0.0024	0.0108	>0.9999
sPD-L1	30.32 (27.02-32.46)	31.93 (24.15-32.89)	27.10 (20.39-29.67)	28.57 (26.93-29.56)	1.74 (1.66-1.85)	1.79 (1.68-1.85)	>0.9999	>0.9999	>0.9999	<0.0001	<0.0001	>0.9999	>0.9999	<0.0001	<0.0001	>0.9999	0.0002	0.0021	0.0008	0.0041	>0.9999
sCTLA-4	7.47 (6.43-9.34)	7.68 (6.18-7.93)	6.13 (5.16-7.11)	7.77 (7.24-8.02)	4.68 (4.41-5.10)	4.68 (4.41-4.98)	>0.9999	0.2325	>0.9999	<0.0001	<0.0001	>0.9999	>0.9999	0.0004	0.0010	0.6371	0.0731	0.1276	0.0003	0.0006	>0.9999
sCD86	3.80 (3.49-4.08)	4.12 (3.88-4.34)	4.44 (3.43-5.14)	4.68 (4.03-5.21)	6.05 (4.99-6.49)	5.76 (5.43-6.41)	>0.9999	0.9793	0.5331	<0.0001	<0.0001	>0.9999	>0.9999	0.0005	0.0010	>0.9999	0.0046	0.0093	0.2378	0.2797	>0.9999

sCD200	32.21 (30.51-36.03)	35.76 (33.66-37.35)	29.77 (27.08-31.87)	31.66 (28.62-33.69)	4.07 (3.84-4.44)	4.07 (3.84-4.33)	>0.9999	>0.9999	>0.9999	<0.0001	<0.0001	0.0814	>0.9999	<0.0001	<0.0001	>0.9999	0.0012	0.0032	0.0015	0.0029	>0.9999
sCD200R	34.09 (30.34-36.44)	35.51 (31.98-36.26)	30.90 (28.55-32.48)	29.72 (26.25-34.52)	3.60 (3.40-3.93)	3.60 (3.40-3.83)	>0.9999	>0.9999	>0.9999	<0.0001	<0.0001	0.9743	>0.9999	<0.0001	<0.0001	>0.9999	0.0002	0.0008	0.0073	0.0124	>0.9999
qPD-1	4.46 (0.47-9.42)	4.73 (0.71-8.02)	8.48 (0.90-18.92)	10.05 (7.53-34.83)	0.88 (0.37-3.61)	0.74 (0.41-1.07)	>0.9999	>0.9999	>0.9999	0.8476	>0.9999	>0.9999	>0.9999	>0.9999	>0.9999	>0.9999	0.1329	0.3721	0.0203	0.0589	>0.9999
qPD-L1	2.13 (0.72-3.66)	1.82 (1.04-3.08)	3.78 (0.60-9.15)	16.80 (6.70-104.50)	0.82 (0.55-1.47)	0.75 (0.51-1.83)	>0.9999	>0.9999	0.2243	>0.9999	>0.9999	>0.9999	0.2323	>0.9999	>0.9999	0.6337	>0.9999	>0.9999	0.0101	0.0151	>0.9999
qCTLA-4	0.95 (0.45-2.21)	0.55 (0.07-1.81)	1.13 (0.43-4.10)	8.85 (2.85-10.38)	0.93 (0.62-1.99)	0.76 (0.26-2.51)	>0.9999	>0.9999	0.0422	>0.9999	>0.9999	0.5568	0.0022	>0.9999	>0.9999	0.4076	>0.9999	>0.9999	0.1069	0.0579	>0.9999

qCD86	0.68 (0.14-3.46)	0.63 (0.22-3.00)	1.04 (0.45-3.72)	10.63 (2.83-25.37)	0.79 (0.40-3.03)	0.82 (0.41-1.92)	>0.9999	>0.9999	0.0138	>0.9999	>0.9999	>0.9999	0.0544	>0.9999	>0.9999	0.3171	>0.9999	>0.9999	0.2096	0.0919	>0.9999
qCD200	7.15 (2.19-27.17)	6.20 (1.67-17.53)	18.32 (2.81-37.79)	6.13 (2.64-36.14)	0.80 (0.59-2.53)	1.21 (0.32-2.10)	>0.9999	>0.9999	>0.9999	0.1347	0.0513	>0.9999	>0.9999	0.4544	0.1864	>0.9999	0.0382	0.0142	0.2876	0.1197	>0.9999
qCD200R	2.79 (0.86-67.68)	3.86 (0.67-60.04)	4.91 (2.34-26.80)	16.34 (11.78-41.22)	1.48 (0.56-2.23)	0.61 (0.28-3.16)	>0.9999	>0.9999	>0.9999	0.4358	0.2700	>0.9999	>0.9999	0.7358	0.4592	>0.9999	0.0572	0.0374	0.0321	0.0206	>0.9999

Supplementary Materials Table S4-Statistically significant correlations between clinical and immunological parameters in patients with IgAN (Spearman rank analysis)

Pair of variables	R	t(N-2)	p
CD8+PD-L1+ & CD4+CD200R+	-0.768	-6.355	0.000
Urea & eGFR	-0.640	-4.408	0.000
TRIGLYCERIDES & HDL	-0.635	-4.347	0.000
CD4+CD200+ & qCD200R	-0.585	-3.816	0.001
Creatine & eGFR	-0.554	-3.520	0.001
CD8+PD-1+ & CD4+CD200R+	-0.539	-3.383	0.002
CD8+PD-1+ & CD4+CD86+	-0.517	-3.194	0.003
Uric acid & eGFR	-0.510	-3.140	0.004
NEU & CD8+PD-1+	-0.510	-3.134	0.004
IgG & Proteinuria	-0.498	-3.042	0.005
Wiek & sCD200	-0.497	-3.031	0.005
CD3-CD16+CD56+PD-1+ & CD4+CD200+	-0.496	-3.025	0.005

CD19+PD-L1+ & CD4+CD200R+	-0.495	-3.014	0.005
HGB & sCD200R	-0.489	-2.969	0.006
NEU & CD19+PD-1+	-0.476	-2.864	0.008
HGB & IgM	-0.462	-2.758	0.010
CD8+PD-1+ & CD4+CD200+	-0.460	-2.740	0.011
RBC & sCD200R	-0.459	-2.731	0.011
HDL & qCTLA-4	-0.451	-2.677	0.012
CD8+PD-L1+ & CD8+CD200R+	-0.447	-2.647	0.013
BAS & sPD-L1	-0.447	-2.647	0.013
HGB & PLT	-0.437	-2.573	0.016
EOS & CD19+CD86+	-0.436	-2.560	0.016
MON & CD19+PD-1+	-0.434	-2.546	0.017
IgG & qCD86	-0.433	-2.540	0.017
Albumin & sCD200R	-0.426	-2.490	0.019
CD19+CD86+ & CD8+CD200+	-0.425	-2.485	0.019
LYM & HDL	-0.421	-2.457	0.020
IgM & CD19+PD-1+	-0.421	-2.456	0.021
CD19+PD-L1+ & CD19+CD200R+	-0.419	-2.441	0.021
CD8+CTLA-4+ & sPD-1	-0.415	-2.414	0.023
Albumin & qCD86	-0.414	-2.408	0.023
CD19+CD86+ & CD8+CD200R+	-0.413	-2.400	0.023
LYM & CD3-CD16+CD56+CD200+	-0.410	-2.381	0.024
CD3-CD16+CD56+PD-L1+ & CD8+CD200+	-0.409	-2.373	0.025
Albumin & sPD-L1	-0.409	-2.373	0.025
CD3-CD16+CD56+CD200R+ & qPD-L1	-0.409	-2.372	0.025
RBC & PLT	-0.409	-2.368	0.025
CD19+PD-1+ & qCD200	-0.404	-2.337	0.027
CD8+CD200+ & sPD-L1	-0.401	-2.317	0.028
CD8+CD200+ & sPD-1	-0.400	-2.310	0.028
CD8+CD86+ & sCD86	-0.397	-2.287	0.030
RBC & IgM	-0.397	-2.286	0.030
CD19+PD-1+ & CD4+CD200R+	-0.393	-2.261	0.032
TRIGLYCERIDES & CD4+CTLA-4+	-0.392	-2.256	0.032

Total protein & qCD86	-0.391	-2.251	0.032
eGFR & IgM	-0.389	-2.237	0.033
qPD-L1 & qCD200	-0.389	-2.235	0.034
Wiek & CD4+CTLA-4+	-0.387	-2.222	0.035
CD4+CD200+ & sPD-L1	-0.382	-2.187	0.037
LDL & sPD-1	-0.381	-2.180	0.038
Creatine & sPD-L1	-0.377	-2.154	0.040
CD4+CTLA-4+ & CD8+CD200R+	-0.375	-2.143	0.041
TRIGLYCERIDES & CD3-CD16+CD56+CD86+	-0.372	-2.122	0.043
Albumin & Proteinuria	-0.372	-2.119	0.043
Total protein & CD3-CD16+CD56+PD-L1+	-0.370	-2.110	0.044
Total protein & Proteinuria	-0.365	-2.076	0.047
CD3-CD16+CD56+CD200+ & qCD200	-0.364	-2.069	0.048
HDL & IgA	-0.364	-2.068	0.048
BAS & CD4+CTLA-4+	-0.364	-2.068	0.048
NEU & CD19+PD-L1+	-0.364	-2.068	0.048
HDL & CD19+CD200R+	-0.362	-2.053	0.049
CD8+PD-1+ & CD19+PD-L1+	0.362	2.052	0.050
PLT & sCD200R	0.363	2.059	0.049
TRIGLYCERIDES & qCD86	0.364	2.066	0.048
qPD-1 & qCTLA-4	0.365	2.072	0.048
Creatine & CD4+CD200+	0.365	2.075	0.047
NEU & PLT	0.366	2.078	0.047
PLT & Proteinuria	0.366	2.080	0.047
LDL & CD4+CD200+	0.366	2.082	0.047
HGB & Total protein	0.368	2.096	0.045
CD8+CTLA-4+ & CD4+CD86+	0.369	2.099	0.045
CD19+PD-L1+ & CD19+CD86+	0.370	2.105	0.044
CD4+PD-L1+ & sCD86	0.372	2.121	0.043
CD3-CD16+CD56+PD-L1+ & sPD-1	0.372	2.122	0.043
CD4+CD200R+ & CD8+CD200R+	0.373	2.128	0.042
CD8+CTLA-4+ & CD19+CTLA-4+	0.374	2.131	0.042
sPD-1 & sPD-L1	0.375	2.142	0.041

CHOLESTEROL & Proteinuria	0.379	2.165	0.039
CD19+CTLA-4+ & CD8+CD86+	0.380	2.175	0.038
WBC & NEU	0.382	2.188	0.037
Wiek & IgM	0.383	2.196	0.037
Urea & Albumin	0.384	2.203	0.036
WBC & CD3-CD16+CD56+PD-L1+	0.385	2.207	0.036
NEU & Proteinuria	0.386	2.214	0.035
LYM & CD4+CD86+	0.388	2.229	0.034
Wiek & CD8+CD200R+	0.390	2.243	0.033
RBC & CD4+CD200R+	0.393	2.264	0.032
CD3-CD16+CD56+CD200+ & qCD86	0.394	2.266	0.031
Proteinuria & qCD86	0.399	2.301	0.029
CD19+PD-1+ & CD4+CTLA-4+	0.399	2.305	0.029
Albumin & CD8+CD200+	0.400	2.308	0.029
WBC & sCD200R	0.411	2.386	0.024
EOS & sCD200R	0.411	2.387	0.024
LYM & HGB	0.421	2.455	0.021
HGB & eGFR	0.426	2.489	0.019
Creatine & CD8+CD200+	0.427	2.501	0.018
CD3-CD16+CD56+CD200+ & CD8+CD200R+	0.427	2.502	0.018
CD3-CD16+CD56+CTLA-4+ & CD4+CD86+	0.430	2.519	0.018
IgM & Proteinuria	0.433	2.542	0.017
WBC & Proteinuria	0.437	2.574	0.016
NEU & IgM	0.440	2.589	0.015
WBC & IgM	0.444	2.622	0.014
LYM & RBC	0.445	2.626	0.014
CD4+CTLA-4+ & CD8+CTLA-4+	0.452	2.679	0.012
WBC & sCD200	0.454	2.695	0.012
qPD-1 & qCD200R	0.455	2.700	0.012
sPD-L1 & qCD86	0.458	2.723	0.011
qPD-L1 & qCD86	0.466	2.787	0.009
CD19+PD-1+ & CD19+PD-L1+	0.475	2.855	0.008
IgM & sCD200R	0.475	2.860	0.008

BAS & HGB	0.480	2.893	0.007
EOS & Uric acid	0.480	2.893	0.007
CD19+PD-1+ & CD8+CD200+	0.497	3.035	0.005
EOS & TRIGLYCERIDES	0.500	3.056	0.005
Creatine & Albumin	0.510	3.138	0.004
CD8+PD-1+ & CD8+PD-L1+	0.511	3.143	0.004
RBC & eGFR	0.516	3.185	0.004
CD3-CD16+CD56+CD200R+ & qCD200	0.532	3.326	0.002
LDL & CD19+CTLA-4+	0.543	3.421	0.002
CD19+PD-1+ & CD8+PD-L1+	0.561	3.584	0.001
CD8+PD-L1+ & CD19+PD-L1+	0.564	3.612	0.001
CD4+CTLA-4+ & CD19+CTLA-4+	0.601	3.975	0.000
Creatine & Uric acid	0.609	4.058	0.000
Urea & Uric acid	0.630	4.289	0.000
IgG & Albumin	0.673	4.815	0.000
Total protein & Albumin	0.718	5.462	0.000
IgG & Total protein	0.793	6.891	0.000
CHOLESTEROL & LDL	0.839	8.154	0.000
RBC & HGB	0.867	9.219	0.000
Urea & Creatine	0.871	9.380	0.000

Supplementary Materials Table S5- Statistically significant correlations between clinical and immunological parameters in patients with MPGN (Spearman rank analysis)

Pair of variables	R	t(N-2)	p
BAS & CD19+CD200R+	-0.581	-3.781	0.001
CD8+CD200+ & qCTLA-4	-0.573	-3.699	0.001
Urea & HDL	-0.566	-3.629	0.001
CD19+PD-L1+ & CD19+CD86+	-0.562	-3.596	0.001
CD3-CD16+CD56+CTLA-4+ & CD4+CD86+	-0.546	-3.449	0.002
PLT & Urea	-0.541	-3.405	0.002
WBC & Total protein	-0.531	-3.320	0.003
PLT & Albumin	-0.525	-3.264	0.003

CD4+PD-L1+ & CD19+CD86+	-0.516	-3.184	0.004
RBC & CD19+CTLA-4+	-0.505	-3.092	0.004
MON & sPD-L1	-0.505	-3.092	0.004
LYM & RBC	-0.495	-3.014	0.005
CD4+CD86+ & CD8+CD200+	-0.479	-2.884	0.007
HDL & Albumin	-0.462	-2.759	0.010
CD8+CD200R+ & sPD-L1	-0.448	-2.654	0.013
CD19+PD-L1+ & CD8+CD200+	-0.446	-2.638	0.013
HDL & CD8+PD-1+	-0.437	-2.570	0.016
Total protein & sPD-L1	-0.429	-2.513	0.018
CHOLESTEROL & Albumin	-0.429	-2.512	0.018
HDL & CD3-CD16+CD56+PD-1+	-0.428	-2.509	0.018
RBC & IgM	-0.427	-2.495	0.019
Albumin & sPD-1	-0.425	-2.486	0.019
WBC & Urea	-0.424	-2.477	0.020
RBC & sPD-L1	-0.424	-2.477	0.020
CD8+CD200+ & sCD86	-0.417	-2.431	0.022
HDL & Total protein	-0.415	-2.415	0.023
CD8+PD-L1+ & CD19+CD86+	-0.411	-2.388	0.024
Uric acid & sPD-L1	-0.408	-2.362	0.025
HGB & sPD-L1	-0.404	-2.338	0.027
RBC & CD8+CTLA-4+	-0.404	-2.337	0.027
EOS & RBC	-0.398	-2.293	0.030
Uric acid & CD19+CTLA-4+	-0.394	-2.269	0.031
CD3-CD16+CD56+CD200+ & CD3-CD16+CD56+CD200R+	-0.393	-2.259	0.032
CD3-CD16+CD56+CD86+ & sCTLA-4	-0.390	-2.243	0.033
PLT & sCD200R	-0.390	-2.241	0.033
Wiek & CD19+CD200R+	-0.386	-2.217	0.035
CD4+PD-L1+ & CD8+CD200+	-0.384	-2.201	0.036
CD19+CD200+ & CD8+CD200R+	-0.380	-2.172	0.038
IgG & qCD200	-0.376	-2.146	0.041
CD3-CD16+CD56+CTLA-4+ & qCTLA-4	-0.374	-2.131	0.042
MON & qCD86	-0.373	-2.128	0.042

Albumin & qCD200	-0.371	-2.111	0.044
CHOLESTEROL & CD3-CD16+CD56+PD-1+	-0.370	-2.110	0.044
RBC & Proteinuria	-0.370	-2.107	0.044
WBC & Albumin	-0.369	-2.102	0.045
CHOLESTEROL & CD19+CD200+	-0.369	-2.098	0.045
CD3-CD16+CD56+PD-L1+ & qPD-L1	-0.365	-2.074	0.047
WBC & CD3-CD16+CD56+PD-L1+	-0.365	-2.073	0.047
CD19+CD200+ & CD19+CD200R+	-0.365	-2.072	0.048
CD19+PD-1+ & CD19+CD86+	-0.364	-2.070	0.048
Uric acid & qCD200	-0.363	-2.062	0.049
RBC & CD19+PD-1+	-0.362	-2.058	0.049
Creatine & CD8+CD86+	-0.362	-2.055	0.049
CD4+PD-1+ & CD8+PD-1+	0.366	2.080	0.047
CD3-CD16+CD56+CTLA-4+ & CD8+CD200+	0.366	2.084	0.046
MON & Creatine	0.369	2.101	0.045
LDL & Proteinuria	0.369	2.104	0.045
qCD86 & qCD200R	0.370	2.104	0.044
CD19+CTLA-4+ & CD4+CD200R+	0.372	2.119	0.043
MON & Uric acid	0.374	2.132	0.042
Urea & IgA	0.374	2.133	0.042
CD4+CTLA-4+ & CD19+CD200+	0.375	2.143	0.041
PLT & sPD-1	0.379	2.166	0.039
sPD-L1 & sCD200R	0.379	2.169	0.039
LDL & qCD200R	0.380	2.172	0.038
sPD-1 & sPD-L1	0.380	2.172	0.038
Total protein & CD19+CD86+	0.383	2.195	0.037
qPD-L1 & qCD86	0.383	2.196	0.037
CD4+PD-1+ & CD3-CD16+CD56+PD-L1+	0.383	2.196	0.037
Wiek & sCD86	0.384	2.197	0.036
Creatine & Total protein	0.385	2.209	0.036
Wiek & IgG	0.386	2.211	0.035
LYM & CHOLESTEROL	0.392	2.257	0.032
CHOLESTEROL & IgM	0.394	2.267	0.031

MON & CD3-CD16+CD56+PD-1+	0.397	2.288	0.030
PLT & CD19+CD86+	0.397	2.290	0.030
Albumin & CD8+CTLA-4+	0.399	2.301	0.029
WBC & sCTLA-4	0.400	2.307	0.029
EOS & CD19+CTLA-4+	0.400	2.313	0.028
LYM & IgG	0.401	2.313	0.028
CD8+PD-1+ & CD3-CD16+CD56+PD-L1+	0.401	2.315	0.028
WBC & PLT	0.401	2.318	0.028
EOS & Albumin	0.402	2.322	0.028
BAS & eGFR	0.406	2.351	0.026
CD4+CTLA-4+ & sCD200R	0.409	2.371	0.025
Creatine & Uric acid	0.413	2.397	0.023
HGB & TRIGLYCERIDES	0.418	2.435	0.022
WBC & sPD-L1	0.425	2.483	0.019
NEU & Uric acid	0.425	2.486	0.019
RBC & qPD-L1	0.426	2.491	0.019
TRIGLYCERIDES & Proteinuria	0.436	2.564	0.016
CD8+PD-1+ & CD19+PD-1+	0.438	2.579	0.015
qPD-1 & qCD200R	0.439	2.585	0.015
EOS & CD8+CTLA-4+	0.444	2.626	0.014
Wiek & MON	0.445	2.627	0.014
BAS & CD19+CD200+	0.445	2.629	0.014
EOS & IgA	0.448	2.651	0.013
IgA & Total protein	0.454	2.694	0.012
HDL & sCD86	0.455	2.707	0.011
CHOLESTEROL & Proteinuria	0.459	2.731	0.011
HDL & LDL	0.464	2.768	0.010
LYM & sCTLA-4	0.464	2.769	0.010
CD4+CTLA-4+ & CD8+CTLA-4+	0.466	2.787	0.009
CD4+CTLA-4+ & CD4+CD200R+	0.469	2.810	0.009
CD4+PD-1+ & CD4+PD-L1+	0.470	2.817	0.009
Proteinuria & CD3-CD16+CD56+CD200R+	0.470	2.820	0.009
NEU & qCD200R	0.475	2.853	0.008

CD4+CD200+ & CD8+CD200+	0.478	2.876	0.008
qPD-L1 & qCD200R	0.483	2.915	0.007
EOS & BAS	0.485	2.935	0.007
CD8+PD-1+ & CD4+CD200R+	0.485	2.937	0.007
Uric acid & IgG	0.492	2.992	0.006
CD4+CTLA-4+ & CD19+CTLA-4+	0.496	3.026	0.005
CD8+CTLA-4+ & CD19+CD200+	0.497	3.031	0.005
TRIGLYCERIDES & CD3-CD16+CD56+CTLA-4+	0.498	3.041	0.005
PLT & HDL	0.500	3.053	0.005
Urea & Total protein	0.500	3.054	0.005
CD8+PD-1+ & CD4+PD-L1+	0.504	3.089	0.004
IgG & Albumin	0.509	3.127	0.004
Urea & Creatine	0.510	3.138	0.004
CHOLESTEROL & TRIGLYCERIDES	0.512	3.155	0.004
sCD86 & qCTLA-4	0.529	3.301	0.003
Total protein & CD3-CD16+CD56+PD-L1+	0.532	3.324	0.002
CD4+CD86+ & qCTLA-4	0.532	3.325	0.002
CHOLESTEROL & LDL	0.534	3.339	0.002
CD4+PD-1+ & CD19+PD-L1+	0.537	3.370	0.002
CD8+PD-1+ & CD19+PD-L1+	0.538	3.378	0.002
CD19+PD-1+ & CD4+PD-L1+	0.538	3.381	0.002
Urea & Albumin	0.566	3.637	0.001
CD4+PD-1+ & CD19+PD-1+	0.567	3.646	0.001
CD8+CTLA-4+ & CD19+CTLA-4+	0.570	3.671	0.001
IgA & Albumin	0.577	3.741	0.001
Total protein & Albumin	0.603	4.004	0.000
TRIGLYCERIDES & LDL	0.624	4.221	0.000
IgG & Total protein	0.638	4.386	0.000
CHOLESTEROL & HDL	0.672	4.802	0.000
CD19+PD-1+ & CD19+PD-L1+	0.715	5.406	0.000
CD4+PD-L1+ & CD19+PD-L1+	0.775	6.493	0.000

Supplementary Materials Table S6 - Receiver Operating Characteristic (ROC) curve analysis for determining the sensitivity and specificity of the parameters studied and their ability to distinguish individual disease entities from each other and from healthy volunteers.

	CD4+PD-1+		
	IgAN vs. MPGN	IgAN vs. HV	MPGN vs. HV
Area	0.6167	1	1
Std. Error	0.07405	0	0
95% confidence interval	0,4715 to 0,7618	1,000 to 1,000	1,000 to 1,000
P value	0.1206	<0,0001	<0,0001
	CD8+PD-1+		
	IgAN vs. MPGN	IgAN vs. HV	MPGN vs. HV
Area	0.6433	0.9706	0.9667
Std. Error	0.07228	0.02167	0.01917
95% confidence interval	0,5017 to 0,7850	0,9281 to 1,000	0,9291 to 1,000
P value	0.0565	<0,0001	<0,0001
	CD19+PD-1+		
	IgAN vs. MPGN	IgAN vs. HV	MPGN vs. HV
Area	0.5011	0.9544	0.9189
Std. Error	0.07784	0.02262	0.03302
95% confidence interval	0,3486 to 0,6537	0,9101 to 0,9988	0,8542 to 0,9836
P value	0.9882	<0,0001	<0,0001
	CD3-CD16+CD56+PD-1+		
	IgAN vs. MPGN	IgAN vs. HV	MPGN vs. HV
Area	0.7039	0.7644	0.9639
Std. Error	0.06645	0.06902	0.0195
95% confidence interval	0,5737 to 0,8341	0,6292 to 0,8997	0,9257 to 1,000
P value	0.0067	0.0004	<0,0001
	CD4+CTLA-4+		
	IgAN vs. MPGN	IgAN vs. HV	MPGN vs. HV

Area	0.7	0.8589	0.9333
Std. Error	0.06959	0.05137	0.04554
95% confidence interval	0,5636 to 0,8364	0,7582 to 0,9596	0,8441 to 1,000
P value	0.0078	<0,0001	<0,0001
	CD8+CTLA-4+		
	IgAN vs. MPGN	IgAN vs. HV	MPGN vs. HV
Area	0.7111	0.8122	0.87
Std. Error	0.07169	0.06017	0.06063
95% confidence interval	0,5706 to 0,8516	0,6943 to 0,9302	0,7512 to 0,9888
P value	0.005	<0,0001	<0,0001
	CD19+CTLA-4+		
	IgAN vs. MPGN	IgAN vs. HV	MPGN vs. HV
Area	0.6311	0.6422	0.7583
Std. Error	0.07455	0.07343	0.06886
95% confidence interval	0,4850 to 0,7772	0,4983 to 0,7861	0,6234 to 0,8933
P value	0.0811	0.0584	0.0006
	CD3-CD16+CD56+CTLA-4+		
	IgAN vs. MPGN	IgAN vs. HV	MPGN vs. HV
Area	0.7428	0.8017	0.6133
Std. Error	0.06389	0.0591	0.07372
95% confidence interval	0,6176 to 0,8680	0,6858 to 0,9175	0,4688 to 0,7578
P value	0.0012	<0,0001	0.1316
	CD4+CD200+		
	IgAN vs. MPGN	IgAN vs. HV	MPGN vs. HV
Area	0.6322	1	1
Std. Error	0.07515	0	0
95% confidence interval	0,4849 to 0,7795	1,000 to 1,000	1,000 to 1,000
P value	0.0785	<0,0001	<0,0001

	CD8+CD200+		
	IgAN vs. MPGN	IgAN vs. HV	MPGN vs. HV
Area	0.76	1	1
Std. Error	0.06281	0	0
95% confidence interval	0,6369 to 0,8831	1,000 to 1,000	1,000 to 1,000
P value	0.0005	<0,0001	<0,0001
	CD19+CD200+		
	IgAN vs. MPGN	IgAN vs. HV	MPGN vs. HV
Area	0.5344	1	1
Std. Error	0.07665	0	0
95% confidence interval	0,3842 to 0,6847	1,000 to 1,000	1,000 to 1,000
P value	0.6467	<0,0001	<0,0001
	CD3-CD16+CD56+CD200+		
	IgAN vs. MPGN	IgAN vs. HV	MPGN vs. HV
Area	0.5256	0.9594	0.9533
Std. Error	0.07554	0.02215	0.02428
95% confidence interval	0,3775 to 0,6736	0,9160 to 1,000	0,9058 to 1,000
P value	0.7338	<0,0001	<0,0001
	CD4+PD-L1+		
	IgAN vs. MPGN	IgAN vs. HV	MPGN vs. HV
Area	0.7167	0.9989	1
Std. Error	0.06838	0.001882	0
95% confidence interval	0,5826 to 0,8507	0,9952 to 1,000	1,000 to 1,000
P value	0.0039	<0,0001	<0,0001
	CD8+PD-L1+		
	IgAN vs. MPGN	IgAN vs. HV	MPGN vs. HV
Area	0.8356	0.9372	0.9978
Std. Error	0.05919	0.03862	0.003051
95% confidence interval	0,7195 to 0,9516	0,8615 to 1,000	0,9918 to 1,000

P value	<0,0001	<0,0001	<0,0001
	CD19+PD-L1+		
	IgAN vs. MPGN	IgAN vs. HV	MPGN vs. HV
Area	0.7478	0.9628	0.955
Std. Error	0.06896	0.02276	0.02539
95% confidence interval	0,6126 to 0,8829	0,9182 to 1,000	0,9052 to 1,000
P value	0.001	<0,0001	<0,0001
	CD3-CD16+CD56+PD-L1+		
	IgAN vs. MPGN	IgAN vs. HV	MPGN vs. HV
Area	0.645	0.5022	0.6489
Std. Error	0.07154	0.07567	0.07143
95% confidence interval	0,5048 to 0,7852	0,3539 to 0,6505	0,5089 to 0,7889
P value	0.0537	0.9764	0.0476
	CD4+CD86+		
	IgAN vs. MPGN	IgAN vs. HV	MPGN vs. HV
Area	0.5461	0.8878	0.8678
Std. Error	0.07535	0.04523	0.04969
95% confidence interval	0,3984 to 0,6938	0,7991 to 0,9764	0,7704 to 0,9652
P value	0.5395	<0,0001	<0,0001
	CD8+CD86+		
	IgAN vs. MPGN	IgAN vs. HV	MPGN vs. HV
Area	0.5067	0.8733	0.8711
Std. Error	0.0758	0.04974	0.05197
95% confidence interval	0,3581 to 0,6552	0,7758 to 0,9708	0,7692 to 0,9730
P value	0.9293	<0,0001	<0,0001
	CD19+CD86+		
	IgAN vs. MPGN	IgAN vs. HV	MPGN vs. HV

Area	0.6006	1	1
Std. Error	0.07446	0	0
95% confidence interval	0,4546 to 0,7465	1,000 to 1,000	1,000 to 1,000
P value	0.1809	<0,0001	<0,0001
	CD3-CD16+CD56+CD86+		
	IgAN vs. MPGN	IgAN vs. HV	MPGN vs. HV
Area	0.5489	0.65	0.6239
Std. Error	0.07503	0.0716	0.07281
95% confidence interval	0,4018 to 0,6960	0,5097 to 0,7903	0,4812 to 0,7666
P value	0.5154	0.0459	0.0993
	CD4+CD200R+		
	IgAN vs. MPGN	IgAN vs. HV	MPGN vs. HV
Area	0.5022	1	1
Std. Error	0.07555	0	0
95% confidence interval	0,3542 to 0,6503	1,000 to 1,000	1,000 to 1,000
P value	0.9764	<0,0001	<0,0001
	CD8+CD200R+		
	IgAN vs. MPGN	IgAN vs. HV	MPGN vs. HV
Area	0.5667	1	1
Std. Error	0.07849	0	0
95% confidence interval	0,4128 to 0,7205	1,000 to 1,000	1,000 to 1,000
P value	0.375	<0,0001	<0,0001
	CD19+CD200R+		
	IgAN vs. MPGN	IgAN vs. HV	MPGN vs. HV
Area	0.5633	0.9533	0.9289
Std. Error	0.07523	0.02467	0.03167
95% confidence interval	0,4159 to 0,7108	0,9050 to 1,000	0,8668 to 0,9910
P value	0.3994	<0,0001	<0,0001

	CD3-CD16+CD56+CD200R+		
	IgAN vs. MPGN	IgAN vs. HV	MPGN vs. HV
Area	0.8383	0.9617	0.7178
Std. Error	0.05007	0.02072	0.06569
95% confidence interval	0,7402 to 0,9365	0,9211 to 1,000	0,5890 to 0,8465
P value	<0,0001	<0,0001	0.0038
	sPD-1		
	IgAN vs. MPGN	IgAN vs. HV	MPGN vs. HV
Area	0.7106	1	1
Std. Error	0.06712	0	0
95% confidence interval	0,5790 to 0,8421	1,000 to 1,000	1,000 to 1,000
P value	0.0051	<0,0001	<0,0001
	sPD-L1		
	IgAN vs. MPGN	IgAN vs. HV	MPGN vs. HV
Area	0.7278	1	1
Std. Error	0.06703	0	0
95% confidence interval	0,5964 to 0,8592	1,000 to 1,000	1,000 to 1,000
P value	0.0024	<0,0001	<0,0001
	qPD-1		
	IgAN vs. MPGN	IgAN vs. HV	MPGN vs. HV
Area	0.6	0.6844	0.7817
Std. Error	0.07551	0.07078	0.06278
95% confidence interval	0,4520 to 0,7480	0,5457 to 0,8232	0,6586 to 0,9047
P value	0.1833	0.0141	0.0002
	qPD-L1		
	IgAN vs. MPGN	IgAN vs. HV	MPGN vs. HV
Area	0.6494	0.6233	0.7306
Std. Error	0.07377	0.07506	0.06788
95% confidence interval	0,5048 to 0,7940	0,4762 to 0,7704	0,5975 to 0,8636

P value	0.0468	0.1008	0.0022
	sCTLA-4+		
	IgAN vs. MPGN	IgAN vs. HV	MPGN vs. HV
Area	0.6589	0.9567	0.8711
Std. Error	0.07017	0.02188	0.0465
95% confidence interval	0,5214 to 0,7964	0,9138 to 0,9996	0,7800 to 0,9622
P value	0.0345	<0,0001	<0,0001
	sCD86+		
	IgAN vs. MPGN	IgAN vs. HV	MPGN vs. HV
Area	0.6967	0.9611	0.8583
Std. Error	0.06871	0.021	0.04631
95% confidence interval	0,5620 to 0,8313	0,9200 to 1,000	0,7676 to 0,9491
P value	0.0089	<0,0001	<0,0001
	qCTLA-4+		
	IgAN vs. MPGN	IgAN vs. HV	MPGN vs. HV
Area	0.7189	0.58	0.6761
Std. Error	0.06613	0.07461	0.07003
95% confidence interval	0,5893 to 0,8485	0,4338 to 0,7262	0,5389 to 0,8134
P value	0.0036	0.2871	0.0191
	qCD86+		
	IgAN vs. MPGN	IgAN vs. HV	MPGN vs. HV
Area	0.6944	0.5689	0.6372
Std. Error	0.06744	0.07615	0.07237
95% confidence interval	0,5623 to 0,8266	0,4196 to 0,7181	0,4954 to 0,7791
P value	0.0097	0.3593	0.0679
	sCCD200+		

	IgAN vs. MPGN	IgAN vs. HV	MPGN vs. HV
Area	0.7878	1	1
Std. Error	0.05718	0	0
95% confidence interval	0,6757 to 0,8999	1,000 to 1,000	1,000 to 1,000
P value	0.0001	<0,0001	<0,0001
	sCD200R+		
	IgAN vs. MPGN	IgAN vs. HV	MPGN vs. HV
Area	0.765	1	1
Std. Error	0.06025	0	0
95% confidence interval	0,6469 to 0,8831	1,000 to 1,000	1,000 to 1,000
P value	0.0004	<0,0001	<0,0001
	qCD200+		
	IgAN vs. MPGN	IgAN vs. HV	MPGN vs. HV
Area	0.5678	0.8033	0.7733
Std. Error	0.07558	0.06095	0.0686
95% confidence interval	0,4196 to 0,7159	0,6839 to 0,9228	0,6389 to 0,9078
P value	0.3671	<0,0001	0.0003
	qCD200R+		
	IgAN vs. MPGN	IgAN vs. HV	MPGN vs. HV
Area	0.5556	0.6967	0.8517
Std. Error	0.07943	0.06856	0.04781
95% confidence interval	0,3999 to 0,7112	0,5623 to 0,8310	0,7580 to 0,9454
P value	0.4598	0.0089	<0,0001

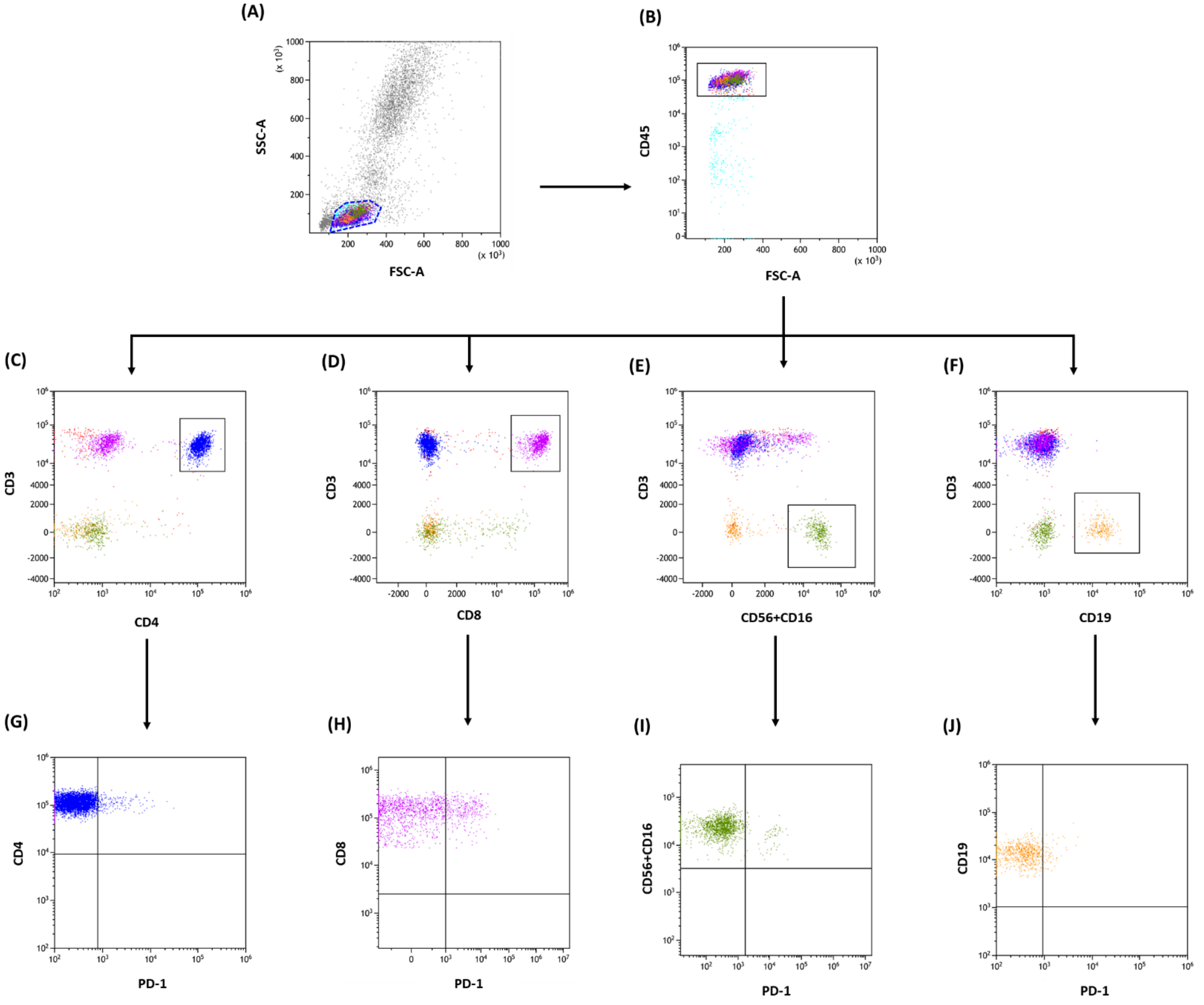

Figure S1 - Example of a gating strategy for IgA nephropathy and the PD-1 molecule. The figure shows the gating strategy used in the flow cytometric analysis. In the first step (A), a lymphocyte population was selected based on the FSC and SSC dispersion parameters, selecting cells with appropriate size and granularity. Then (B), CD45+ cells were isolated from this population, which allowed us to limit the analysis to leukocytes. In the next step (C–F), the main lymphocyte subpopulations were separated: CD3+CD4+ T cells (C), CD3+CD8+ T cells (D), NK cells defined as CD3–CD56+CD16+ (E), and B lymphocytes identified as CD3–CD19+ (F). In the last step (G–J), the expression of the PD-1 receptor was assessed in each of the distinguished subpopulations: CD4+ T lymphocytes, CD8+ T lymphocytes, NK cells and B lymphocytes, respectively. The entire procedure enabled a detailed analysis of PD-1 expression in various fractions of immune cells.

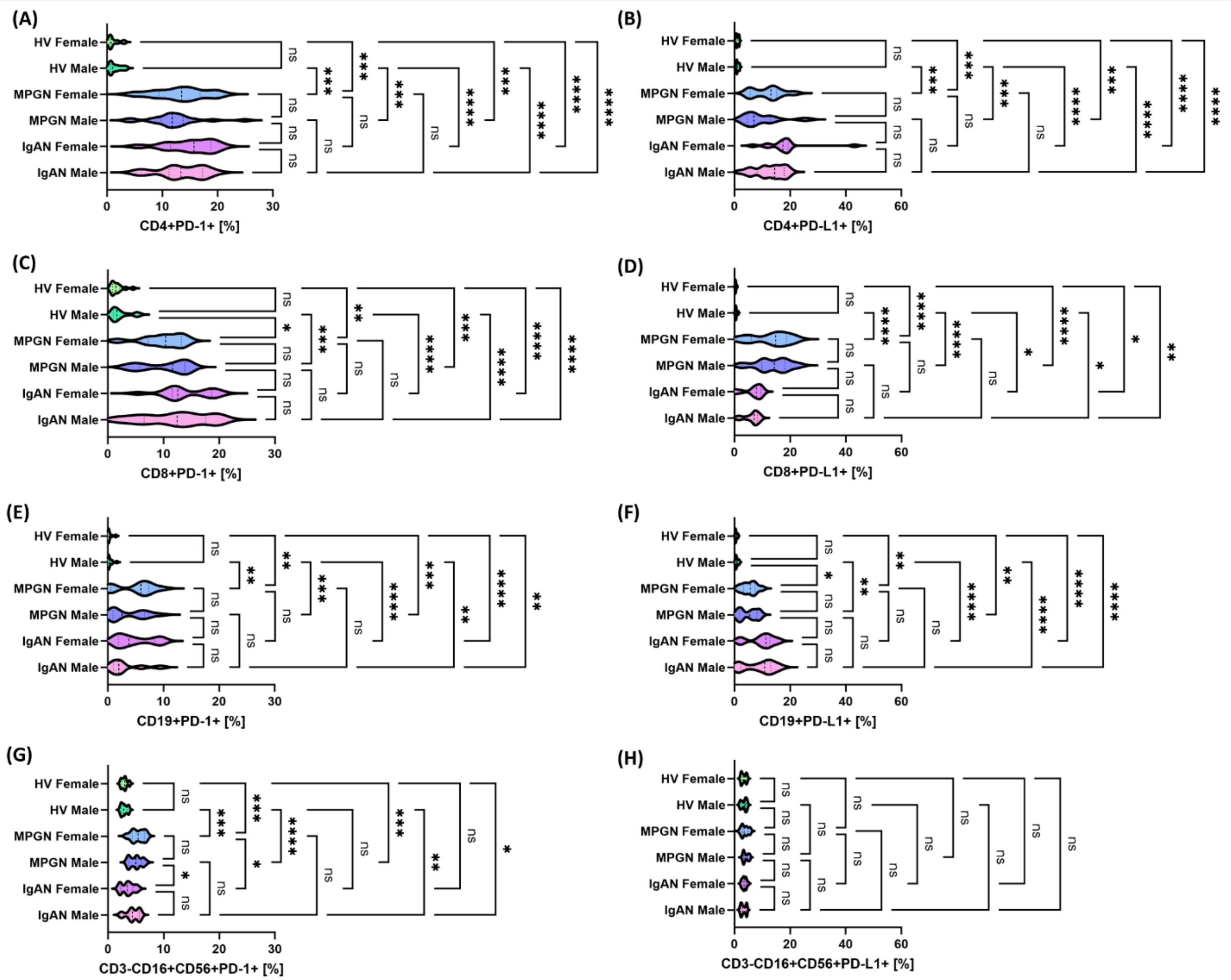

Figure S2. Expression of PD-1 and PD-L1 on selected immune cell subpopulations in patients with IgA nephropathy (IgAN, purple), MPGN (blue), and healthy volunteers (HV, green) by gender. (A) CD4+PD-1+, (B) CD4+PD-L1+, (C) CD8+PD-1+, (D) $CD8^{+}PD\text{-}L1^{+}$, (E) CD19+PD-1+, (F) CD19+PD-L1+, (G) CD3-CD16+CD56+PD-1+, (H) CD3-CD16+CD56+PD-L1+. Data are presented in the form of violin plots, illustrating the median, quartiles, and the full distribution of values in individual groups (HV women/men, IgAN women/men, MPGN women/men). Statistical differences between groups are marked: *p < 0.05; **p < 0.01; ***p < 0.001; ****p < 0.0001; ns – no significance.

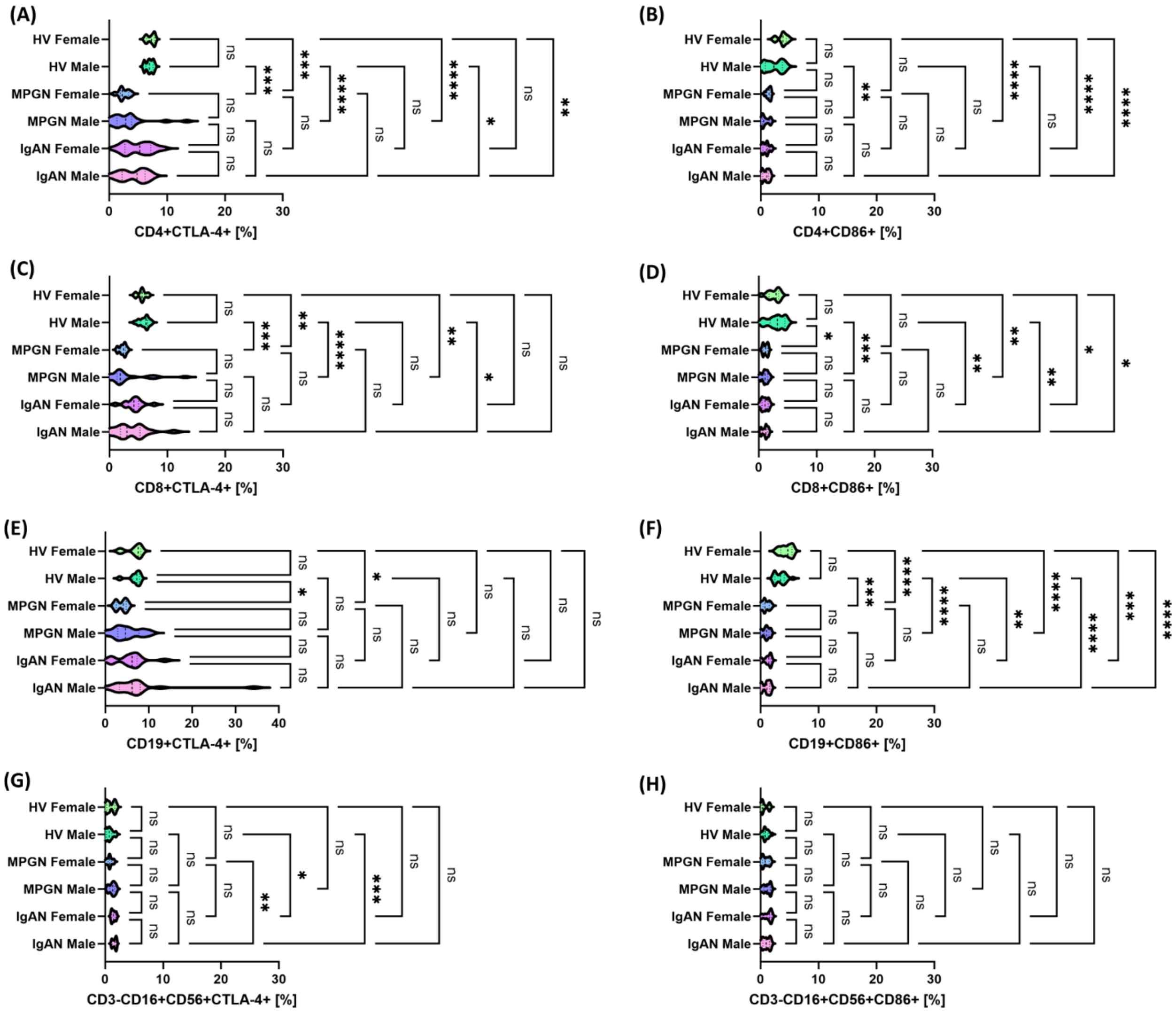

Figure S3. Expression of CTLA-4 and CD86 molecules on selected immune cell subpopulations, categorized by gender in healthy controls (HV, green), IgAN (purple), and MPGN (blue). (A) CD4+CTLA-4+, (B) CD4+CD86+, (C) CD8+CTLA-4+, (D) CD8+CD86+, (E) CD19+CTLA-4+, (F) CD19+CD86+, (G) CD3-CD16+CD56+CTLA-4+, (H) CD3-CD16+CD56+CD86+. Data were presented as violin plots, showing the median, quartiles, and the full distribution of values in the study groups, taking into account gender (women/men). Statistical differences were marked: *p < 0.05; **p < 0.01; ***p < 0.001; ****p < 0.0001; ns – no significance.

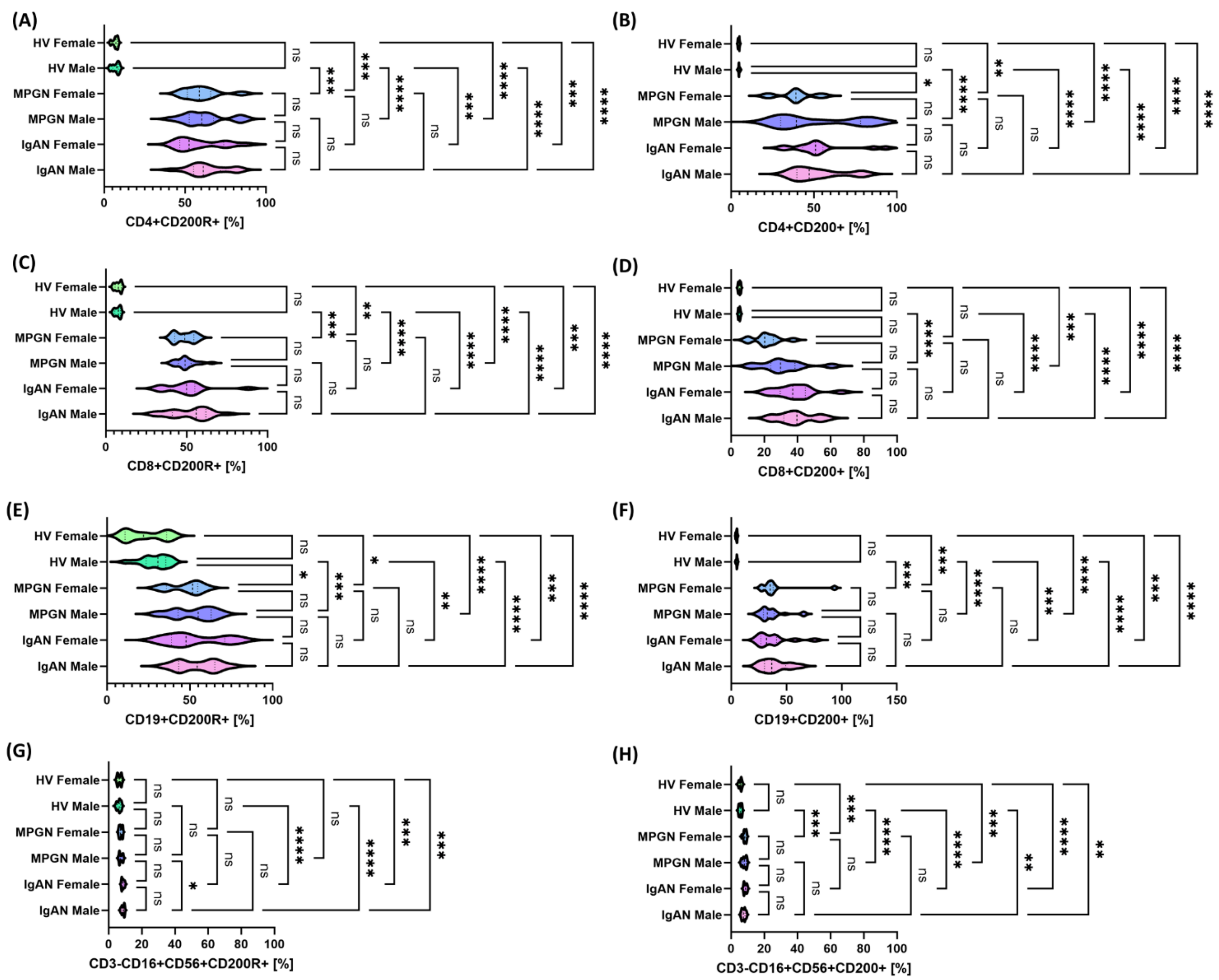

Figure S4. Expression of CD200R and CD200 molecules on the surface of selected immune cell subpopulations by gender in healthy volunteers (HV, green), patients with IgAN (purple), and patients with MPGN (blue). (A) CD4+CD200R+, (B) CD4+CD200+, (C) CD8+CD200R+, (D) CD8+CD200+, (E) CD19+CD200R+, (F) CD19+CD200+, (G) CD3-CD16+CD56+CD200R+, (H) CD3-CD16+CD56+CD200+. Data are presented as violin plots, showing the median, quartiles, and the full distribution of values in the study groups (women/men). Statistical differences are marked: *$p < 0.05$; **$p < 0.01$; ***$p < 0.001$; ****$p < 0.0001$; ns – no significance.

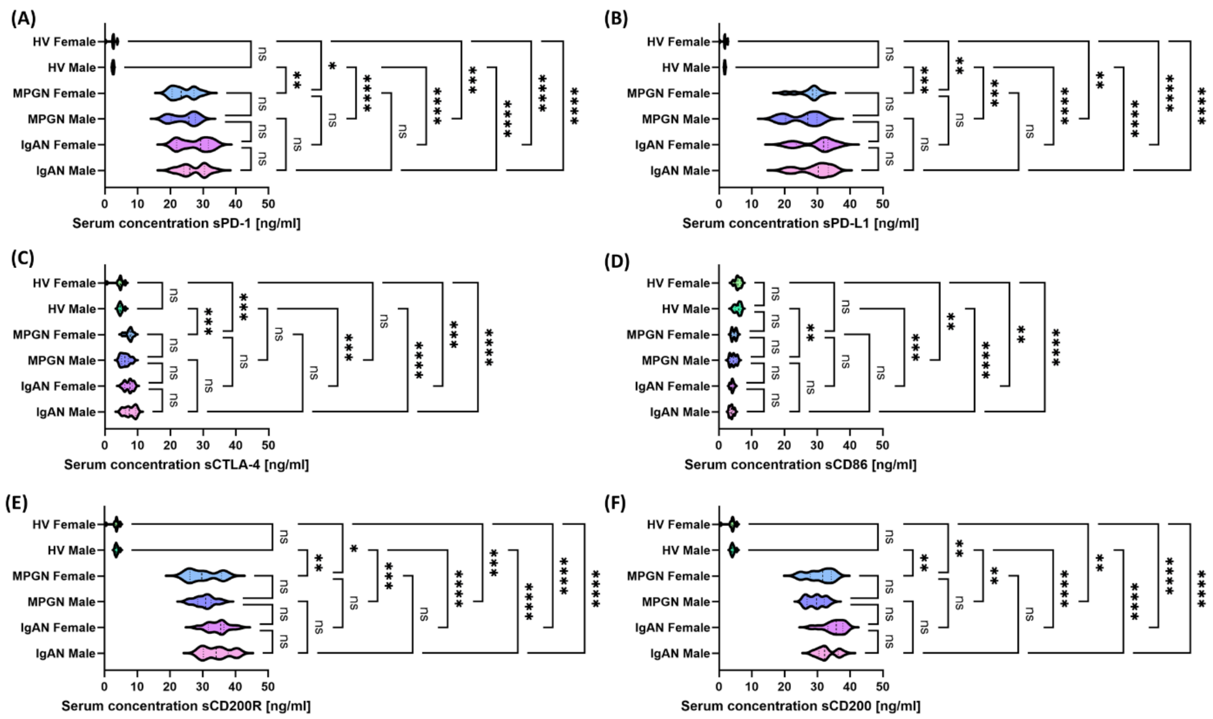

Figure S5. Serum concentrations of soluble immune checkpoints by gender in healthy volunteers (HV, green), patients with IgAN (purple), and patients with MPGN (blue). (A) sPD-1, (B) sPD-L1, (C) sCTLA-4, (D) sCD86, (E) sCD200R, (F) sCD200. Data are presented in violin plots, illustrating the median, quartiles, and the full distribution of values in the study groups, taking into account gender (women/men). Significant differences between groups are indicated by: *p < 0.05; **p < 0.01; ***p < 0.001; ****p < 0.0001; ns – not significant.

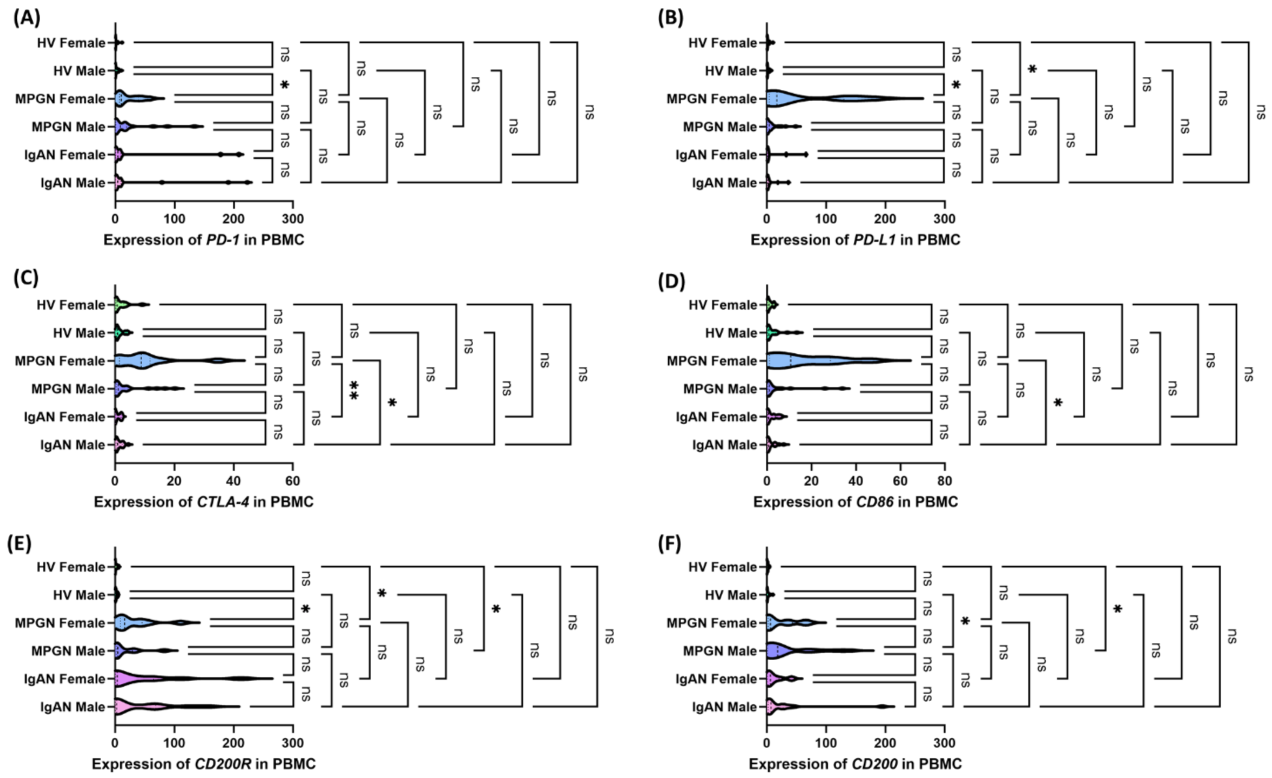

Figure S6. Expression of genes encoding immune checkpoints in peripheral blood mononuclear cells (PBMCs) by gender in patients with IgA nephropathy (IgAN, purple), MPGN (blue), and healthy volunteers (HV, green). (A) PD-1, (B) PD-L1, (C) CTLA-4, (D) CD86, (E) CD200R, (F) CD200. Data are presented as violin plots, including median, quartiles, and the full distribution of values across study groups (women/men). Significant differences between groups are indicated by: *p < 0.05; **p < 0.01; ***p < 0.001; ****p < 0.0001; ns – not significant.
